# Human but not robotic gaze facilitates action prediction

**DOI:** 10.1016/j.isci.2022.104462

**Published:** 2022-05-25

**Authors:** Emmanuele Tidoni, Henning Holle, Michele Scandola, Igor Schindler, Loron Hill, Emily S. Cross

**Affiliations:** 1 Human Technology Laboratory, Department of Psychology, University of Hull, Hull HU6 7RX, UK; 2 Department of Psychology, University of Hull, Hull HU6 7RX, UK; 3 NPSY.Lab.VR & BASIC_NPSY, Department of Human Sciences, University of Verona, 37129 Verona, Italy; 4 Department of Cognitive Science, Macquarie University, Sydney, NSW 2109, Australia; 5 Institute of Neuroscience and Psychology, University of Glasgow, Glasgow G12 8QB, UK

**Keywords:** cognitive neuroscience, Robotics, research methodology social sciences

## Abstract

Do people ascribe intentions to humanoid robots as they would to humans or non-human-like animated objects? In six experiments, we compared people’s ability to extract non-mentalistic (i.e., where an agent is looking) and mentalistic (i.e., what an agent is looking at; what an agent is going to do) information from gaze and directional cues performed by humans, human-like robots, and a non-human-like object. People were faster to infer the mental content of human agents compared to robotic agents. Furthermore, although the absence of differences in control conditions rules out the use of non-mentalizing strategies, the human-like appearance of non-human agents may engage mentalizing processes to solve the task. Overall, results suggest that human-like robotic actions may be processed differently from humans’ and objects’ behavior. These findings inform our understanding of the relevance of an object’s physical features in triggering mentalizing abilities and its relevance for human–robot interaction.

## Highlights

• People differently ascribe mental content to human-like and non-human-like agents
• A human-like shape may automatically engage mentalizing processes
• Human actions are interpreted faster than non-human actions

## Graphical abstract

**Figure:**
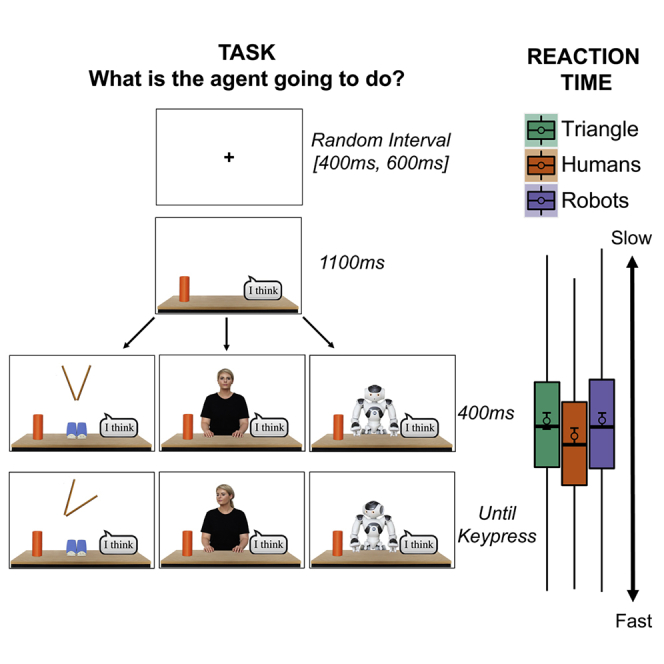

## Introduction

During the past two decades, research examining the cognitive and psychological principles facilitating human–robot interaction for recreational (Palinko et al., 2016), assistive (Melkas et al., 2020), therapeutical (Langer et al., 2019), and educational purposes (Senft et al., 2019) has rapidly increased. Designing autonomous agents whose form and motion are modeled after humans is thought to facilitate the tendency to attribute human qualities to these agents (Fink, 2012; Press, 2011). Furthermore, modeling robots’ behaviors after human social behavior is thought to increase human acceptance. For example, a robot engaging in direct compared to random gaze during a small talk interaction facilitates people’s acceptance of the robot and leads to greater reports of human-likeness (Babel et al., 2021).

The ability to represent others’ mental content from observing their actions is considered crucial for engaging in daily social interactions (Catmur, 2015; Tidoni and Candidi, 2016; Schurz et al., 2020; Heyes and Catmur, 2021). However, comparing the ability to infer others’ mental states from the observation of human and non-human agents’ actions is complicated by several confounding variables known to affect stimuli creation. Robotic and human limbs differ in size and length across different machines, and it is difficult to precisely match human and robot kinematics (e.g., especially creating a mechanical agent with biological motion; Urgen et al., 2013; Bisio et al., 2014; supporting information in Cross et al., 2012). Current literature also suggests that brain regions active when observing robotic actions are affected by the human-like appearance of robots (Saygin et al., 2010,^2012^; Saygin and Stadler, 2012; Urgen et al., 2012; 2013; 2018, 2019; Urgen and Saygin, 2020). Furthermore, the tendency to mimic and the ability to imitate others is affected by the perception of (non) biological motion (Hofree et al., 2015) and by the goal-directedness of the observed robotic act (Bisio et al., 2014), respectively. Moreover, actions carried out to achieve different aims are characterized by different kinematics. For example, people move slightly differently when they aim to deceive an observer compared to acting as normal (i.e., with no intention to deceive; Tidoni et al., 2013; Finisguerra et al., 2018; Makris and Urgesi, 2015), or when they grasp an object to drink compared to pass it to someone (Bianco et al., 2020). Nonetheless, matching the kinematics across different agents and intentions is fundamental to test the participants’ ability to process the hidden states of the observed agent rather than low-level differences during action observation (e.g., the gaze direction of the observed agent; Catmur, 2015; Tidoni and Candidi, 2016; Thompson et al., 2019).

It has been suggested that an observed individual’s head and gaze movements may represent a good proxy to infer their mental content (Becchio et al., 2008). For example, observing a person looking at an object may automatically generate the expectation that this person will grasp that object (look to grasp), or observing a person turning their head and gazing toward another person may indicate a different intent (e.g., look to listen, or to start a conversation). Moreover, the human gaze toward an object of interest typically precedes a subsequent hand movement (Johansson et al., 2001). Thus, using object-directed gaze behavior as a cue to others’ intentions instead of manual actions reduces the kinematic variability across different agents and different intentions and is crucial to advance our understanding of the social perceptual building blocks of human–robot interactions. Indeed, provided that robots will (sooner or later) be taking on assistive and collaborative roles alongside humans in all manner of social environments (including hospitals, schools, care homes, and our homes), it is vital to understand how robot gaze and head movements affect the interaction with a human partner (Admoni et al., 2014;Admoni and Scassellati, 2017; Mutlu et al., 2009; Pan et al., 2020; Strabala et al., 2013; Fiore et al., 2013). For example, establishing how implicit cues (e.g., gaze and head movements) preceding an action performed by an artificial agent are interpreted may be crucial to improve communication in robot-to-human handover tasks (Strabala et al., 2013; Johanson et al., 2020; Ortenzi et al., 2021).

Previous studies have shown that observing an agent gazing toward an object facilitates taking that agent’s perspective (Furlanetto et al., 2013; Ward et al., 2019; but see Quesque et al., 2018, for a general tendency to take a decentered perspective). Similarly, observing robots gazing toward objects increases the degree to which people take their perspective but always to a lesser extent than humans (Zhao et al., 2015; Zhao and Malle, 2022). Furthermore, observing others gazing toward a graspable object recruits brain regions typically active during the execution and observation of actions toward that object (Pierno et al., 2006). ALthough this literature establishes a foundation for understanding how we read other people’s social behavior from their gaze direction, it remains unclear to what extent robotic gaze and head movements toward objects evoke the same processes active during human gaze perception. Moreover, in order to ensure the reliability and broader applicability of such an approach, it is vital for experimental tasks to rule out low-level explanations of experimental findings (e.g., spatial stimulus-response mapping: faster responses for “right” gaze using a right-handed compared to a left-handed response), and to ensure that the observer maintains a distinction between their own and others’ mental states (Quesque and Rossetti, 2020).

Following this logic, through the current study we investigated people’s ability to understand human and robotic gaze behavior by manipulating the task’s demand (e.g., either detecting the non-mentalistic visuo-spatial features of an action or inferring what an agent is going to do next; Catmur, 2015; Tidoni and Candidi, 2016; Thompson et al., 2019) across a series of six independent experiments. Specifically, in experiments 1, 2, and 5, participants detected how an action was performed (i.e., where an agent was looking). We considered such tasks less mentalistic than experiments 3, 4, and 6, because understanding where an agent is looking does not require any reflection about the observed agent’s mental state or visual percept. Moreover, in typical gaze cueing tasks, response times of valid trials (i.e., trials where a target appears in the same spatial location of the observed gaze shift) have been shown to be identical between humans and robots (Wiese et al., 2012; Wykowska et al., 2014; Li et al., 2015). In Experiment 3, participants indicated what the agent was looking at. In experiments 4 and 6, participants indicated what the agent was going to do. We considered experiments 3, 4, and 6 as mentalistic tasks because participants focused on what the agent was seeing (i.e., implying the mental state of seeing; Teufel et al., 2010; Bukowski et al., 2015; Furlanetto et al., 2016) and what the goal of the agent was (i.e., implying the ability to plan goal-directed actions). Hence, we did not measure spontaneous perspective-taking abilities (Cole et al., 2015; Conway et al., 2017) because we explicitly asked participants to focus on different levels of the observed action. Moreover, we controlled the role of the visual form and textures of the observed agents by comparing the observation of human and robotic gaze movement with an object-like directional cue (i.e., a triangle-shaped object without eyes). Finally, because we aimed to assess people’s ability to generally reflect upon human and non-human agents’ mental content from gaze observation (i.e., not merely detecting a directional change in the agent’s gaze or being unable to disengage from a humanoid robot, Chaminade and Okka, 2013), we compared gaze behaviors directed toward graspable and non-graspable objects (i.e., 3D printed geometric shapes, a text bubble), to non-object-directed gazes as control conditions (i.e., looking up or down in experiments 1, 2, 3 and 4, looking down in experiments 5 and 6). Importantly, we presented non-ambiguous objects (e.g., a sphere that does not require any mental rotation to be identified instead of numbers like six or nine typically used in perspective-taking tasks; Surtees et al., 2016; Zhao and Malle, 2022) before the agent appeared, and the observed agent was always front-facing participants (i.e., reducing the need of mental rotation to understand the posture of the agent). Moreover, we created the impression of an apparent motion (Shiffrar and Freyd, 1990; Schenke et al., 2016) by presenting two images in rapid succession, and asked participants to pay attention to the observed action. Hence, we think that our tasks and trial timeline can be interpreted within an action observation framework because our design differs from typical visuo-spatial perspective-taking tasks (dynamic stimuli, objects presented before the agent, fixed agent orientation, participants’ attention to the agent’s action).

## Results

**Figure 1:**
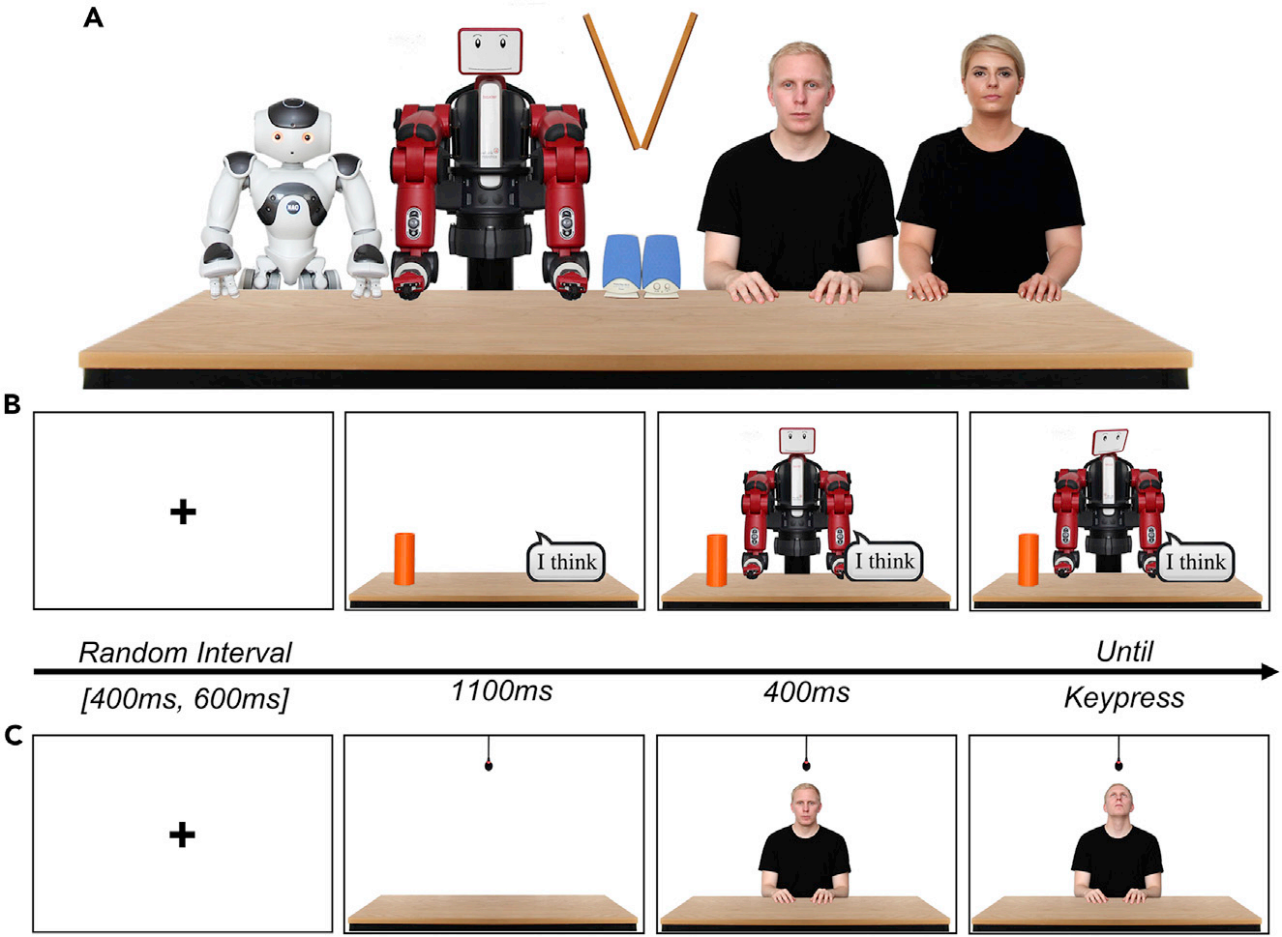
The set of agents and Trial Timeline (A) Agents are displayed side by side for graphical purposes. During the experiments, agents were always centered to screen. The triangle shaped object was presented with a set of speakers to suggest its capacity to emit sounds. (B) An example trial timeline: after a variable interval the scenario is displayed followed by the presentation of the agent looking straight for 400ms before gazing toward one direction (in the image an example of the agent looking toward the text bubble). (C) In experiments 5 and 6, the graspable objects and the text bubble were replaced by a microphone. See STAR Methods and Video S1 for further details.

We performed six separate experiments. Some experiments were conducted to explore alternative hypothesis and corroborate data interpretation. The actual order in which the experiments were conducted was Experiment 3, 2, 4, 6, 5, and 1. The order of the experiments as presented in this manuscript is changed for logical consistency. Participants observed an agent appearing behind a table and gazing toward different directions (participants’ left, right, up or down) and objects (experiments 1, 2, 3 and 4: graspable objects, and a text bubble; for experiments 5 and 6: a microphone; see Apparatus and Task and Video S1 for further details). This setup allowed to present multiple gaze behaviors and resembles typical visual perspective-taking tasks (Furlanetto et al., 2013; Zhao et al., 2015; Quesque et al., 2018). Moreover, by changing task demands, we were able to test whether focusing participants’ attention to low-level features of the observed gaze or reflecting upon the hidden states of the agent yields different results. Specifically, participants indicated where the agent was looking (experiments 1, 2, and 5), why the agent was looking in a specific direction (experiments 4 and 6), and what the agent was looking at (Experiment 3). Agents were two human actors (one male, one female), two humanoid robots (NAO, Softbank Robotics; Baxter, Rethink Robotics), and a portable lectern edited to resemble a triangle-shaped object with realistic visual textures. Stimuli of humans and humanoid robots had their trunk and upper arms visible and were edited to give the impression that their upper limbs were resting on the table (Figure 1A). The faces of Baxter were created from an open-source database (Fitter and Kuchenbecker, 2016) and the support column behind Baxter’s screen was removed to avoid any distraction from its face (see Figure S1 comparing the original and the edited image).

Video S1. Instructions, Stimuli, and Trial Timeline, related to Figure 1 In this video we provide a visual description of the instructions and the stimuli used to create the gaze and head apparent motion for all six experiments. The video also shows an example of the trial timeline for Experiment 1-4 and 5-6.

At the end of each experiment, participants rated their exposure to media robotic content (see STAR Methods section).

### Experiment 1– Detecting where an agent is looking (egocentric perspective)

We explored people’s ability to detect where an agent is looking (i.e., its gaze direction). If detecting a change in human gaze is favored by participants, we should expect a main effect of the observed agent but no interactions with gaze direction.

We aimed to collect data from 80 participants and managed to collect 81 individual datasets. The participants were instructed that each agent could look in four directions: to the participants’ left, right, up or down (henceforth labeled ‘up|down’ condition because each participant used the same randomised-across-subjects key to indicate both directions; see STAR Methods). The participants indicated where the agent was looking: toward their left, right, up|down. Thus, participants answered from an egocentric perspective (i.e., if the agent looked toward its right, which corresponds to the participants’ left, the correct answer was “left”). Crucially, participants answered using a set of keys (‘n’, ‘j’, ‘i’) that were orthogonal for left-right answers. Keys were randomized across the participants.

We removed trials where the response times (RTs) were deemed to be too fast or too slow based on preregistered criteria (4.64%; see STAR Method section for the statistical approach). Then, trials with RTs falling above or below 2.5SD of the overall mean within each block of each participant were removed (2.75%). Three participants with a performance accuracy rate of <65% were removed. Finally, four participants with a performance (either Accuracy or RT) above or below 2.5SD of the overall mean across conditions of the remaining participants were excluded (final sample n = 74). To facilitate comparisons across experiments 1, 2, 3 and 4, “right” and “left” throughout the study and in the figures always refer to the participants’ right and left (i.e., observed agent’s left and right, respectively).

#### Main task performance

**Figure 2:**
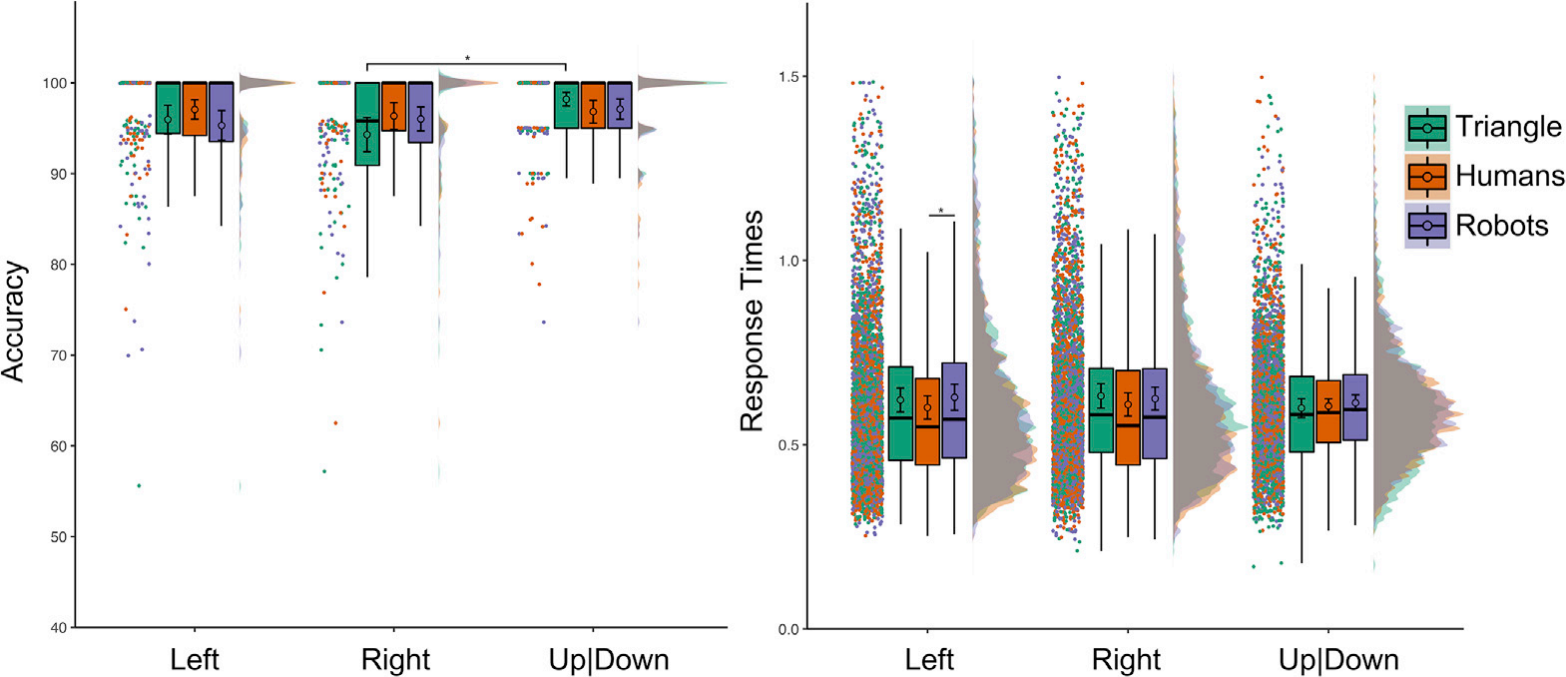
Results of Experiment 1 Accuracy percentage is shown on the left, Response Times expressed in seconds on the right. To provide a comprehensive overview of collected data, raw data from each experimental condition are visualized as raincloud plots, median bar plots (with lower and upper hinges corresponding to the 25^th^ and 75^th^ percentile and whiskers extending no further than 1.5 ∗ “InterQuartile Range” from the hinge), and probability density. The circles inside each median bar plot indicate the average of the by-subject mean-aggregated data for that condition. Error bars represent 95% confidence intervals of the mean based on subject-aggregated data. Data visualization has been possible by adapting the open-source R code “RainCloudPlots” (Allen et al., 2019). The labels “Left” and “Right” indicate the participant’s left and right respectively. Asterisks denote the significant differences (p < 0.05) for both the MLM and the ANOVA on mean-aggregated data as reported in the main text.

We analyzed Accuracy and RTs (see Figure 2) with Agent (human, robot, triangle) and Gaze (left, right, up|down) as within-subjects fixed effects of a multilevel linear model (MLM; see Table S1 in Supplementary Information for details on the fixed and random effects structure of all MLMs). In the case of a two-way interaction between Agents and Gaze, we performed 18 multiple paired comparisons of interest. Specifically, we compared the three gaze directions within each Agent (e.g., right gaze-human agent versus left gaze-human agent; nine comparisons), and each gaze across the three agents (e.g., right gaze-human agent versus right gaze-robot agent; nine comparisons). We also performed a confirmatory ANOVA on mean-aggregated data to support the main analyses. Non-conclusive findings (i.e., less robust, more fragile) are highlighted whenever the MLM and the analyses on mean-aggregated data yield discordant results (see STAR Method sections for further details).

For accuracy, we observed a main effect of Gaze, χ^2^(2) = 10.069, p_MLM_ = 0.007, no main effect of Agent, χ^2^(2) = 1.730, p_MLM_ = 0.421, and a significant Gaze by Agent interaction, χ^2^(4) = 11.373, p_MLM_ = 0.023. The latter suggested that participants were more accurate in recognizing when the triangle was looking up|down (average of mean-aggregated data ± SEM of mean-aggregated data; 98.20 ± 0.37%) compared to the participants’ right (94.30 ± 0.94%; p_MLM_< 0.001, |d| = 0.436, BF_10_ = 63.812). No other Bonferroni corrected pvalues were lower than 0.05 for both multiple comparisons computed on the estimates of the simplified MLM and multiple comparisons using pairwise t-tests on aggregated data (p_MLM_> 0.096, p_MultComp_> 0.263, |d| < 0.291, BF_10_< 2.327).

For RT, we removed incorrect answers (3.55%) from the final dataset. We observed a main effect of Agent, F(2,145.7) = 8.396, p_MLM_< 0.001, ηp2 = 0.107, no main effect of Gaze, F(2,146) = 1.330, p_MLM_ = 0.268, ηp2 = 0.018, and a significant Gaze by Agent interaction, F(4, 289.2) = 3.208, p_MLM_ = 0.013, ηp2 = 0.045. The latter suggested that the participants were faster in detecting humans looking to the participants’ left (0.601 ± 0.016 s) compared to robots (0.629 ± 0.018 s; p_MLM_ = 0.004, |d| = 0.382, BF_10_ = 16.533). No other comparisons survived Bonferroni correction (p_MLM_> 0.027, p_MultComp_> 0.063, |d| < 0.351, BF_10_< 8.114; see STAR Methods for data analysis approach).

#### The role of the looked-at objects

To control any influence of the objects the agents were directing their attention to, we re-analysed the same dataset based on what the agent was looking at (i.e., the graspable objects, the text bubble, up|down).

For accuracy, we observed a main effect of Object, χ2(2) = 11.654, p = 0.003, no effect of Agent, χ2(2) = 1.910, p_MLM_ = 0.385, and a significant Object by Agent interaction, χ2(4) = 11.598, p_MLM_ = 0.021. The latter suggested that the participants were more accurate in recognizing when the triangle was looking up|down (98.20 ± 0.37%) compared to the text bubble (94.52 ± 0.87%; p_MLM_< 0.001, |d| = 0.434, BF10 = 60.570). No other comparisons survived Bonferroni correction (p_MLM_> 0.062, p_MultComp_> 0.102, |d| < 0.331, BF10 < 5.287).

For RT, we did not observe an effect of Object, F(2, 146) = 1.387, p_MLM_ = 0.253, ηp2 = 0.019, nor an Object by Agent interaction, F(4, 290.6) = 2.337, p_MLM_ = 0.056, ηp2 = 0.033.

#### Interim discussion experiment 1

Using keys orthogonal for left-right answers successfully reduced any spatial compatibility effect because we did not observe faster RT to gazes toward participant’s right (i.e., “right” answers) than gazes toward participant’s left (i.e., “left” answers). However, we may have seen a stimulus-response mapping depending on task demands and the observed agent. Specifically, we observed that participants had more difficulty when the triangle looked toward the participant’s right (i.e., the agent’s left side) than up|down. The participants were also faster in recognizing a human than a robot gazing toward the participant’s left (i.e., opposite to their responding hand). These findings may suggest that for non-human agents, conflicting spatial features of the stimulus (e.g., an agent gazing toward the participant’s right, corresponding to the agent’s left, and correct answer “right”; agent gazing toward the participant’s left, corresponding to the agent’s right, and correct answer “left”) may have had an influence in coding the correct response.

An alternative explanation for the drop in accuracy for the triangle might be that the shape of the triangle (pointing downwards) favored the expectation of a vertical movement compared to a lateral one, and participants might not have expected the triangle to turn toward the space occupied by their answering hand or toward a social stimulus like the text bubble. Notably, we did not see any difference in accurately detecting gaze direction across agents. Finally, we note that effect sizes were generally small, and the looked-at-object and visual familiarity (see Supplementary Information) did not affect the ability to detect directional cues from different agents.

### Experiment 2– Detecting where an agent is looking (allocentric perspective)

While Experiment 1 investigated the ability to detect others’ gaze from an egocentric perspective, Experiment 2 tested if taking the agent’s point of view may differently affect performance. If the participants use a spatial strategy to solve the task when adopting an allocentric perspective, we should expect similar results to Experiment 1.

We aimed to collect data from 100 participants and managed to collect 96 individual datasets. The participants were instructed that each agent could look in four directions: to its right, left, up or down. The participants were asked to indicate where the agent was looking: toward its left, right, up|down. Thus, participants answered from an allocentric perspective (i.e., if the agent looked toward its right, which corresponds to the participants’ left, the correct answer was “right”).

We removed trials where RTs were too fast or too slow (5.39%). Then, trials with RTs falling above or below 2.5SD of the overall mean within each block of each participant were removed (2.38%). Five participants with a performance below 65% were removed. Finally, seven participants with a performance (either accuracy or RT) above or below 2.5SD of the overall mean across conditions of the remaining participants were excluded from the final sample. Despite this data management approach, one participant had 0% accuracy when the triangle looked to participants’ right and left, suggesting a misunderstanding of the task. Thus, we removed that participant (final sample n = 83).

**Figure 3:**
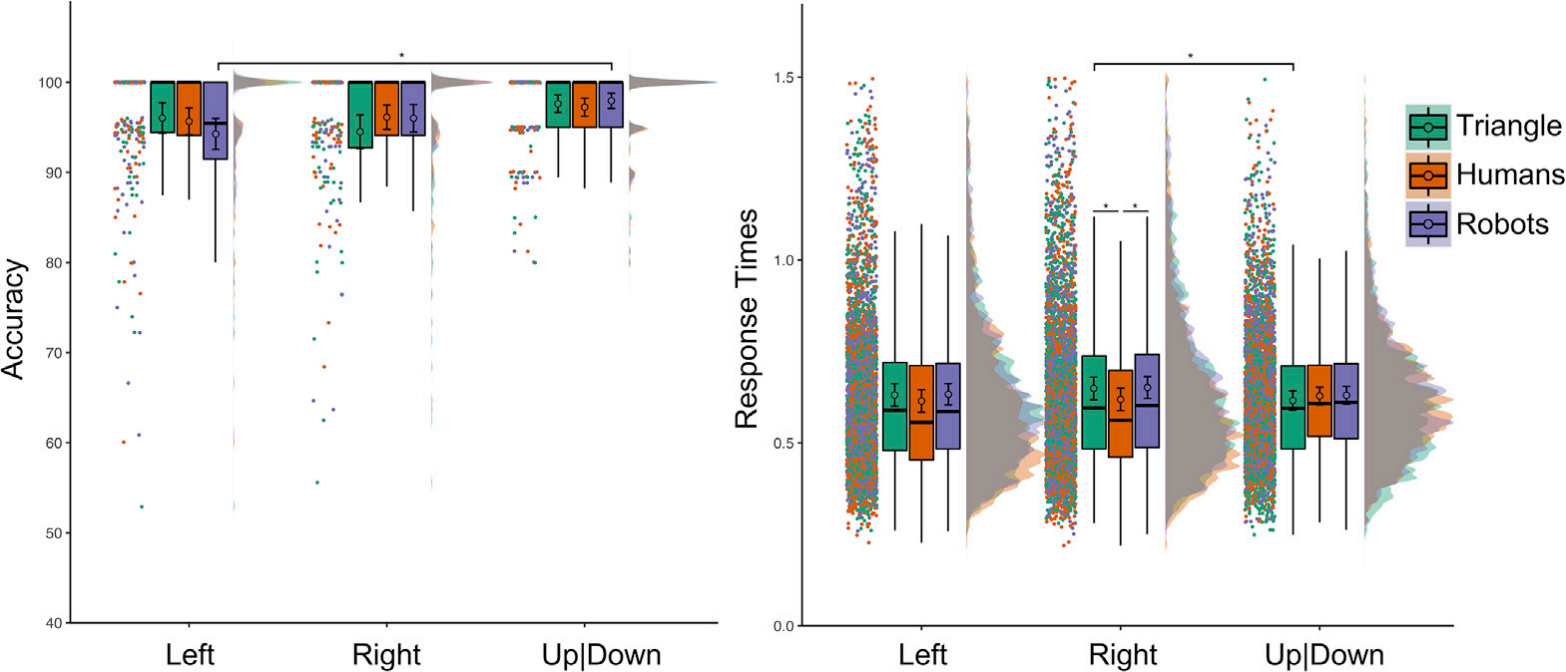
Results of Experiment 2 Accuracy percentage is shown on the left, Response Times expressed in seconds on the right. The labels “Left” and “Right” indicate the participant’s left and right respectively. See Figure 2 for a detailed explanation of our data visualization approach.

We analyzed Accuracy and RTs (see Figure 3) with Agent (human, robot, triangle) and Gaze (left, right, up|down) as within-subject fixed effects. In case of a two-way Agent by Gaze interaction, we performed eighteen multiple paired comparisons of interest as indicated in Experiment 1.

For accuracy, we observed a main effect of Gaze, χ2(2) = 14.867, p_MLM_< 0.001, no main effect of Agent, χ2(2) = 0.044, p_MLM_ = 0.978, and a significant Gaze by Agent interaction, χ2(4) = 11.353, p_MLM_ = 0.023. The latter suggested that participants were more accurate in recognizing when the robot looked up|down (97.96 ± 0.43%) compared to the participants’ left (94.27 ± 0.86%; p_MLM_< 0.001, |d| = 0.462, BF10 = 287.679). No other comparisons survived Bonferroni correction and paired comparisons on aggregated data (p_MLM_> 0.023, p_MultComp_> 0.073, |d| < 0.324, BF10 < 6.776).

For RT, we removed incorrect answers (3.75%) from the final dataset. We observed a main effect of Agent, F(2,486.1) = 11.583, p_MLM_< 0.001, ηp2 = 0.048, no main effect of Gaze, F(2,164) = 1.792, p_MLM_ = 0.170, ηp2 = 0.022, and a significant Gaze by Agent interaction, F(4, 486.1) = 5.726, p_MLM_< 0.001, ηp2 = 0.047. The latter suggested that participants were faster in recognizing the triangle looking up|down (0.616 ± 0.013 s) compared to the participants’ right (0.649 ± 0.016 s; p_MLM_ = 0.048, |d| = 0.348, BF10 = 11.941). The participants were also faster in detecting human agents gazing toward the participants’ right (0.619 ± 0.015 s) compared to the triangle (p_MLM_< 0.001, |d| = 0.447, BF10 = 187.351) and robots (0.651 ± 0.015 s; p_MLM_< 0.001, |d| = 0.494, BF10 = 791.261). No other comparisons survived Bonferroni correction (p_MLM_> 0.088, p_MultComp_> 0.220, |d| < 0.281, BF10 < 2.587).

#### The role of the looked-at objects

To control for any influence of the objects the agents were gazing toward, we analyzed participants’ performance measures based on what the agent was looking at (i.e., the graspable objects, the text bubble, up|down).

For accuracy, we observed a main effect of Object, χ2(2) = 21.010, p_MLM_< 0.001, with participants being more accurate when the agent looked up|down (97.61 ± 0.36%) compared to the graspable object (95.13 ± 0.65%; p_MLM_< 0.001, |d| = 0.383, BF10 = 30.064), and compared to the text bubble (95.70 ± 0.62%; p_MLM_ = 0.001, |d| = 0.301, BF10 = 3.991). We did not observe a main effect of Agent, χ2(2) = 0.033, p_MLM_ = 0.984, nor an Object by Agent interaction, χ2(4) = 8.635, p_MLM_ = 0.071.

For RT, we did not observe an effect of Object, F(2, 163.9) = 0.999, p_MLM_ = 0.371, ηp2 = 0.012. However, we observed a main effect of Agent, F(2, 488.7) = 11.941, p_MLM_< 0.001, ηp2 = 0.049, and an Object by Agent interaction, F(4,488.5) = 5.283, p_MLM_< 0.001, ηp2 = 0.043). The latter revealed that participants were faster when humans looked at the graspable object (0.620 ± 0.016 s) compared to the triangle (0.642 ± 0.016 s; p_MLM_ = 0.012, |d| = 0.347, BF10 = 11.713) and the robot (0.644 ± 0.015 s; p_MLM_ = 0.002, |d| = 0.401, BF10 = 49.026), and when humans looked at the text bubble (0.611 ± 0.014 s) compared to the triangle (0.635 ± 0.015 s; p_MLM_ = 0.004, |d| = 0.404, BF10 = 52.955) and the robot (0.636 ± 0.014 s; p_MLM_ = 0.001, |d| = 0.382, BF10 = 28.772). No other comparisons survived Bonferroni correction (p_MLM_> 0.138, p_MultComp_> 0.184, |d| < 0.289, BF10 < 3.017).

#### Interim discussion experiment 2

Using keys orthogonal for left-right answers successfully reduced any spatial compatibility effect because we did not observe that gazes toward participants’ right (i.e., “left” answers) were faster than gazes toward participants’ left (i.e., “right” answers). However, a stimulus-response mapping interference depending on task demands and the observed agent may explain the data. Specifically, we observed that participants were more accurate in recognizing the robot looking up or down compared to the participants’ left (i.e., right side of the agent). This result mirrors in part the findings of Experiment 1, where we observed the triangle (not the robots) being more accurate when the agent moved up|down compared to the participants’ left. This finding suggests that mental rotation required to solve the task may affect task accuracy depending on the observed agent. Indeed, we also observed faster RT for detecting humans compared to robots and the triangle looking to participant’s right (i.e., agent’s left). This may further suggest that taking the perspective of non-human agents may decrease performance when the spatial features of the stimulus conflict with response mapping (e.g., the agent gazing toward the participant’s left, corresponding to the agent’s right, and correct answer “right”; the agent gazing toward the participant’s right, corresponding to the agent’s left, and correct answer “left”).

Finally, we showed that the objects shown on the table did not affect the participants’ ability to detect agents’ change of gaze (i.e., the differences in detecting agents’ attentional change spread evenly between the two objects), and that visual familiarity did not affect the ability to detect directional cues (see Supplementary Information).

### Experiment 3– Detecting what an agent is looking at

Experiments 1 and 2 indicated that detecting where an agent is directing its attention may be influenced by spatial and social information. The latter was more evident when participants had to take an allocentric perspective of the agent (Experiment 2). Here, we wanted to investigate if similar difficulties are observed during a mentalistic task where participants indicate what the agent sees (e.g., understand others’ visual percepts; Flavell et al., 1981; Bukowski et al., 2015). If detecting what a human agent is looking at is different from detecting their gaze direction, we should expect differences across agents only when they look toward an object but not when they gaze away from it.

We aimed to collect data from 100 participants and managed to collect a total of 101 individual datasets. The participants were instructed that each agent could look at the graspable object, at the text bubble, up or down. The participants were asked to indicate what the agent was looking at (the object, the bubble, up|down).

We removed trials with RTs that were deemed too fast or too slow (3.93%). Then, trials with RTs falling above or below 2.5SD of the overall mean within each block of each participant were removed (1.97%). No participants’ performance was <65%. Finally, six participants had a performance (either accuracy or RT) above or below 2.5SD of the overall mean across conditions of the remaining participants and were excluded from the final sample (n = 95).

#### Main task performance

**Figure 4:**
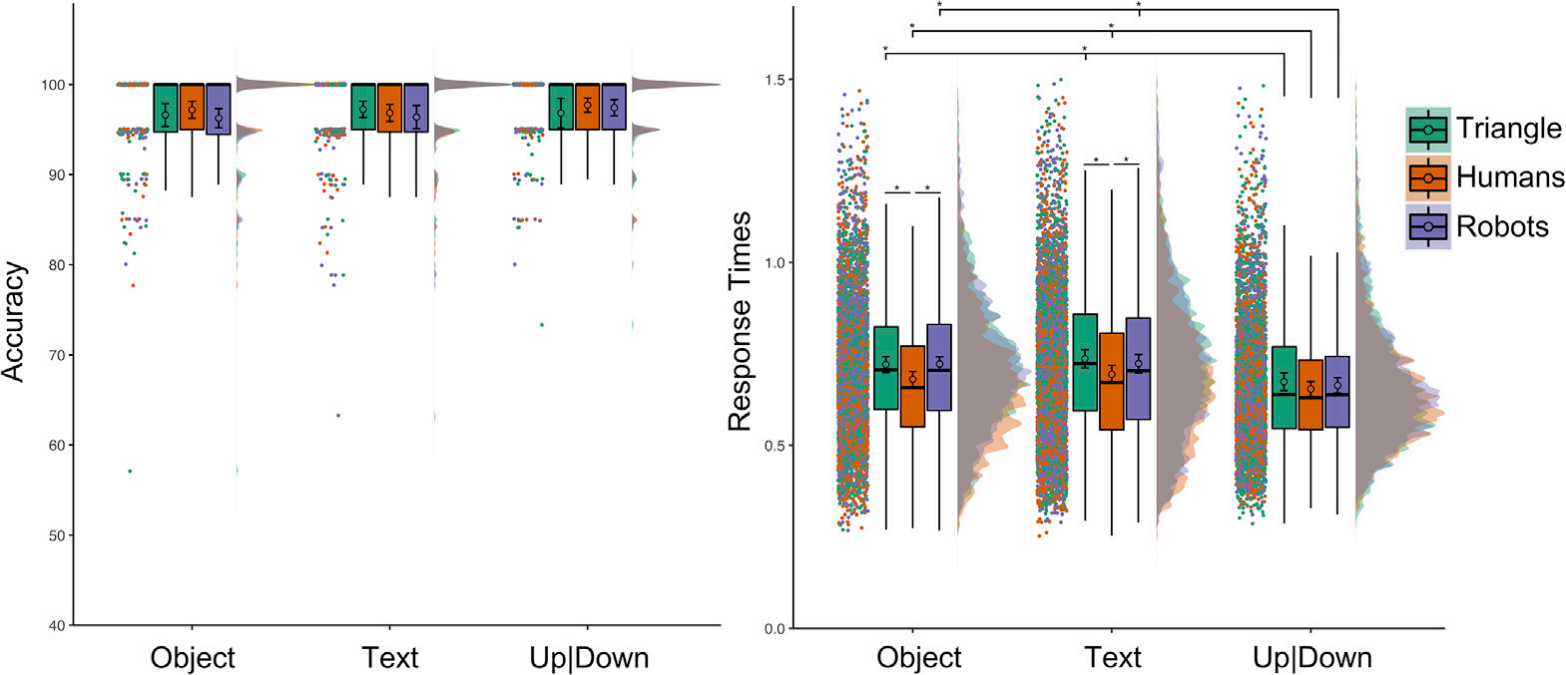
Results of Experiment 3 Accuracy percentage is shown on the left, Response Times expressed in seconds on the right. The labels “Object” and “Text” indicate the conditions where participants detected the agent looking at the graspable object and the text bubble respectively. See Figure 2 for a detailed explanation of our data visualization approach.

We analyzed performance measures (see Figure 4) with Agent (human, robot, triangle) and Object (graspable objects, text bubble, up|down) as within-subject factors. In case of a two-way interaction between Agent and Object, we performed eighteen multiple paired comparisons of interest. Specifically, we compared the three Object levels within each Agent (e.g., graspable object-human agent versus text bubble-human agent; nine comparisons), and each Object level across the three agents (e.g., text bubble-human agent versus text bubble-robot agent; nine comparisons).

For accuracy, we observed no main effects, χ2(2) < 3.133, p_MLM_> 0.209, and no interaction, χ2(4) = 1.948, p_MLM_ = 0.745.

For RT, we removed incorrect answers (3.01%) from the final dataset. We observed a main effect of Agent, F(2,187.6) = 50.888, p_MLM_< 0.001, ηp2 = 0.354, a main effect of Object, F(2,187.9) = 37.257, p_MLM_< 0.001, ηp2 = 0.285, and a significant Agent by Object interaction, F(4, 374.7) = 3.938, p_MLM_ = 0.004, ηp2 = 0.042). The latter suggested that participants were faster in recognizing the triangle looking up|down (0.674 ± 0.012 s) compared to looking at the graspable objects (0.721 ± 0.011 s; p_MLM_< 0.001, |d| = 0.511, BF10 = 5.267e+03) and the text bubble (0.737 ± 0.012 s; p_MLM_< 0.001, |d| = 0.675, BF10 = 3.982e+06). The participants were also faster in recognizing the humans looking up|down (0.654 ± 0.010 s) compared to looking at the graspable objects (0.681 ± 0.011 s; p_MLM_ = 0.017, |d| = 0.360, BF10 = 31.317) and the text bubble (0.694 ± 0.012 s; p_MLM_< 0.001, |d| = 0.440, BF10 = 4.122e+02). The participants were faster to recognize robots looking up|down (0.664 ± 0.011 s) compared to looking at the graspable objects (0.723 ± 0.010 s; p_MLM_< 0.001, |d| = 0.782, BF10 = 4.3363e+08) and the text bubble (0.724 ± 0.013 s; p_MLM_< 0.001, |d| = 0.653, BF10 = 1.599e+06). These differences are not surprising because participants had to remap the answer for right and left gaze each trial as objects location was randomly assigned each trial.

Crucially, participants were faster in detecting human agents looking toward the graspable objects compared to the triangle (p_MLM_< 0.001, |d| = 0.676, BF10 = 4.221e+06) and robots (p_MLM_< 0.001, |d| = 0.667, BF10 = 2.809e+06). The participants were also faster in detecting human agents looking at the text bubble compared to the triangle (p_MLM_< 0.001, |d| = 0.621, BF10 = 4.098e+05) and robots (p_MLM_< 0.001, |d| = 0.451, BF10 = 5.831e+02). No other Bonferroni corrected pvalues were lower than 0.05 for both multiple comparisons computed on the estimates of the simplified MLM and multiple comparisons using pairwise t-tests on aggregated data (p_MLM_> 0.012, p_MultComp_> 0.055, |d| < 0.312, BF10 < 8.342).

#### The role of gaze direction

To control the role of where the agents directed their attention, we analyzed performance measures based on where the agent was looking at (i.e., participants’ right or left, and up|down).

For accuracy, no main effects of visual attention allocation, no interaction with the two main effects, χ2 < 5.067, p_MLM_> 0.231, were observed.

For RT, we observed a main effect of Gaze, F(2,187.6) = 50.814, p_MLM_< 0.001, ηp2 = 0.351, and a significant Agent by Gaze interaction, F(4, 282.3) = 3.614, p_MLM_ = 0.007, ηp2 = 0.038. The latter suggested that participants were faster in recognizing the triangle looking up|down (0.674 ± 0.012 s) compared to looking to participants’ left (0.734 ± 0.011 s; p_MLM_< 0.001, |d| = 0.723, BF10 = 3.216e+07) and right (0.725 ± 0.012 s; p_MLM_< 0.001, |d| = 0.550, BF10 = 2.286e+04). The participants were faster in recognizing the humans looking up|down (0.654 ± 0.010 s) compared to looking to participants’ left (0.698 ± 0.012 s; p_MLM_< 0.001, |d| = 0.550, BF10 = 2.347e+04). The participants were faster in recognizing robots looking up|down (0.664 ± 0.011 s) compared to looking at participants’ left (0.729 ± 0.012 s; p_MLM_< 0.001, |d| = 0.848, BF10 = 8.832e+09) and right (0.716 ± 0.010 s; p_MLM_< 0.001, |d| = 0.633, BF10 = 6.722e+05). Again, these differences are not surprising because participants had to remap the answer for right and left gaze each trial as objects location was randomly assigned each trial.

The participants were faster in detecting human agents looking toward the participants’ left compared to the triangle (p_MLM_< 0.001, |d| = 0.561, BF10 = 3.535e+04) and robots (p_MLM_< 0.001, |d| = 0.467, BF10 = 1.041e+03). We also observed faster RT in detecting human agents looking at the participants’ right compared to the triangle (p_MLM_< 0.001, |d| = 0.767, BF10 = 2.311e+08) and robots (p_MLM_< 0.001, |d| = 0.639, BF10 = 8.714e+05). No other comparisons survived Bonferroni correction (p_MLM_> 0.010, p_MultComp_> 0.055, |d| < 0.333, BF10 < 14.620).

#### Interim discussion experiment 3

The participants were faster in recognizing what human agents were looking at compared to other non-human agents. The results were not affected by the participants’ visual familiarity with robots (see Supplementary Information) or the agent’s gaze direction.

While experiments 1-2 suggested that detecting where a non-human agent is looking at may decrease the ability to map the correct answer with the spatial information derived from the observed movement, asking participants to indicate what the agent is looking to (i.e., focusing on what the agent is seeing) revealed a clearer difference between agents and faster processing of human gaze.

Nonetheless, it may be argued that the participants were simply faster in mapping the observed human action with the correct answer. We note that the participants’ RT was faster for human than non-human agents when they looked at the objects, but not when looking up or down. Thus, matching the correct answer with the observed movement was more difficult for the robots and the triangle in those conditions. This may suggest that trying to represent others’ mental content (i.e., what they are seeing) is harder for non-human compared to human agents. To further explore this idea, participants in experiment 4 completed a similar task to experiment 3 with object locations that did not differ across the entire task.

### Experiment 4– Inferring why an agent is looking at an object

Experiment 3 showed that interpreting what non-human agents are looking at may be more demanding than interpreting what a human sees. Here, we tested whether inferring the forthcoming action of an agent may affect performance when the location of the graspable object and text bubble during the task does not change. In other words, the graspable objects and the text bubble location on the table were fixed for each trial, and participants could, in principle, use a spatial strategy to solve the task (e.g., *the graspable object is always on the agent’s right*, *thus*, *if the agent looks right*, *I will press key ‘n’*). We reasoned that if the participants solved the task by detecting others’ gaze direction, we should expect results similar to experiment 1 or 2. Contrary, if predicting others’ actions from gaze observation requires access to others mental representations, results should mirror the findings of experiment 3.

We aimed to collect data from 80 participants and managed to collect a total of 82 individual datasets. The participants were instructed that each agent could look toward the object to grasp it (gaze to grasp, motor intention), toward the text bubble to speak (gaze to speak, social intention), up or down to do nothing (non-goal directed action as control). For 40 participants, the graspable object was displayed on the agent’s left (with the text bubble on the agent’s right) and, for 42 participants, the graspable object was displayed on the agent’s right (with the text bubble on the agent’s left). The participants were asked to indicate what the agent was going to do (i.e., the agent is going to grasp, to speak, or is looking up or down).

We removed trials whose RTs were too fast or too slow (5.17%). Then, trials whose RTs fell above or below 2.5SD of the overall mean within each block of each participant were removed (2.30%). A further five participants with a performance below 65% were removed. Finally, four participants with a performance (either accuracy or RT) above or below 2.5SD of the overall mean across conditions of the remaining participants were excluded (final sample n = 73; group with the graspable object to the agent’s left n = 36; group with the graspable object to the agent’s right n = 37).

#### Main task performance

**Figure 5:**
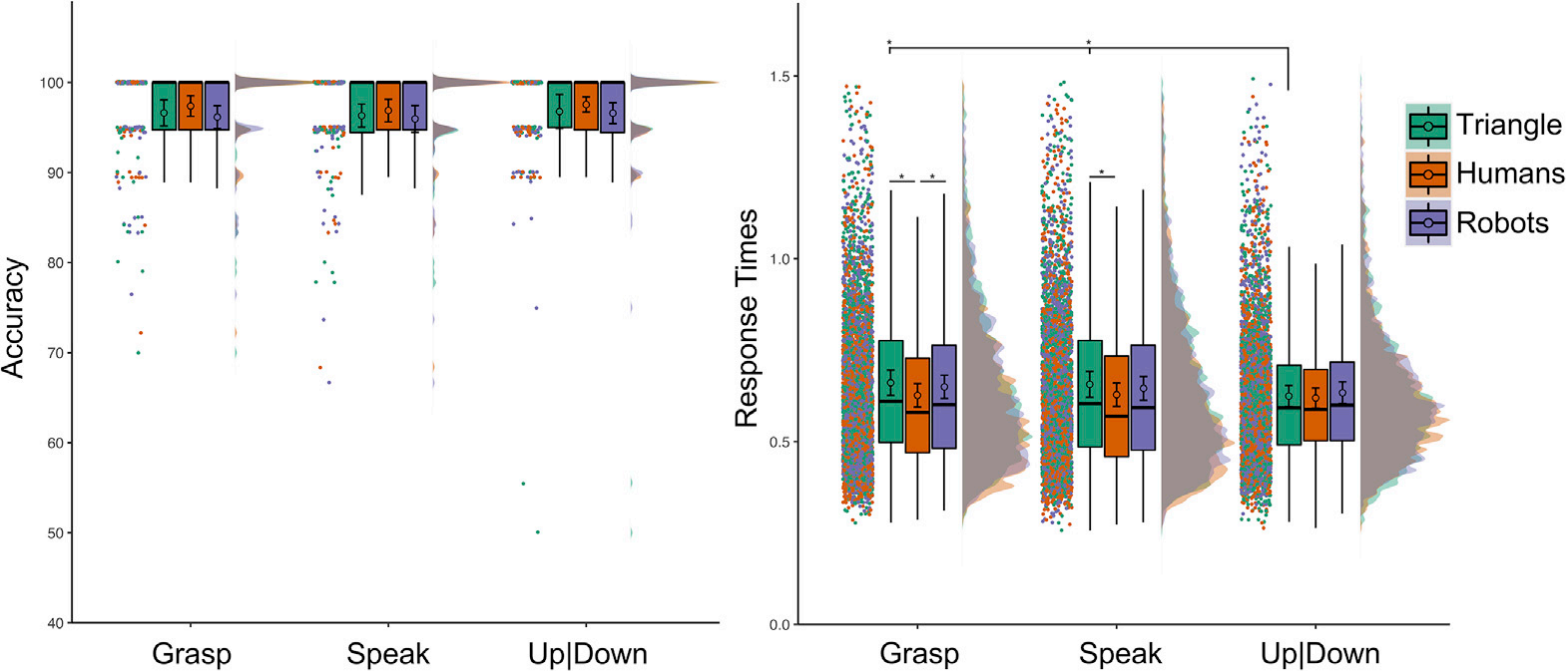
Results of Experiment 4 Accuracy percentage is shown on the left, Response Times expressed in seconds on the right. The labels “Grasp” and “Speak” indicate the condition where participants attributed the intention to grasp and to speak to respectively. See Figure 2 for a detailed explanation of our data visualization approach.

We analyzed performance measures (see Figure 5) with Agent (human, robot, triangle) and Intention (to grasp, to speak, look up|down) as within-subject’s factors, and the graspable object-Location (left, right) as between-subjects factor. In case of a two-way Agent by Intention interaction, we performed eighteen multiple paired comparisons of interest as indicated in experiment 3.

For accuracy, we observed a main effect of Agent, χ2(2) = 6.084, p_MLM_ = 0.048, and a Graspable Object Location by Agent interaction, χ2(2) = 6.158, p_MLM_ = 0.046. These results were not confirmed in the analyses on aggregated data (Agent: F = 2.647, p_Anova_ = 0.074, ηp2 = 0.036; Graspable Object Location by Agent: F = 3.015, p_Anova_ = 0.052, ηp2 = 0.041). No other effects were observed, χ2 < 3.670, p_MLM_> 0.077.

For RT, we removed incorrect answers (3.26%) from the final dataset. We observed a main effect of Agent, F(2,141.7) = 17.296, p_MLM_< 0.001, ηp2 = 0.200, and a significant Agent by Intention interaction, F(4, 282.3) = 3.082, p_MLM_ = 0.017, ηp2 = 0.044. No other main effects or interactions were observed, F < 2.982, p_MLM_> 0.054, ηp2 < 0.040. The two-way interaction suggested that participants were faster in recognizing the triangle looking up|down (0.625 ± 0.015 s) compared to attributing the intention to grasp (0.661 ± 0.017 s; p_MLM_ = 0.010, |d| = 0.412, BF10 = 32.327) and to speak (0.657 ± 0.018 s; p_MLM_ = 0.043, |d| = 0.369, BF10 = 11.684). The participants were also faster in detecting human agents’ intention to grasp (0.627 ± 0.016 s) compared to the triangle (p_MLM_< 0.001, |d| = 0.596, BF10 = 5.877e+03) and robots (0.650 ± 0.016 s; p_MLM_ = 0.009, |d| = 0.414, BF10 = 34.225). Moreover, participants were faster in detecting human agents’ intention to speak (0.629 ± 0.016 s) compared to the triangle (p_MLM_ = 0.001, |d| = 0.486, BF10 = 2.304e+02). No other Bonferroni corrected pvalues were lower than 0.05 for both multiple comparisons computed on the estimates of the simplified MLM and multiple comparisons using pairwise t-tests on aggregated data (p_MLM_> 0.189, p_MultComp_> 0.425, |d| < 0.325, BF10 < 4.471).

#### The role of gaze

To control for gaze direction, we analyzed participants’ performance based on where the agent was looking (i.e., participants’ right or left, and up|down; see Figure S2).

For accuracy, we observed no main effects of visual attention and no interaction with the two main effects, χ2 < 3.157, p_MLM_> 0.206.

For RT, we observed a main effect of Gaze, F(2,142) = 3.117, p_MLM_ = 0.047, ηp2 = 0.042, and a significant Agent by Gaze interaction, F(4, 282.3) = 4.263, p_MLM_ = 0.002, ηp2 = 0.059. No other main effects or interactions with attention were observed, F < 0.3611, p_MLM_> 0.809, ηp2 < 0.005. The two-way interaction suggested that participants were faster in recognizing the triangle looking up|down (0.625 ± 0.015 s) compared to looking to the participants’ right (0.664 ± 0.017 s; p_MLM_ = 0.003, |d| = 0.496, BF10 = 287.824). The participants were also faster in detecting human agents’ looking toward the participants’ left (0.621 ± 0.016 s) compared to the triangle (0.654 ± 0.018 s; p_MLM_< 0.001, |d| = 0.537, BF10 = 9.821e+02) and robots (0.651 ± 0.017 s; p_MLM_< 0.001, |d| = 0.474, BF10 = 1.630e+02). The participants were also faster in detecting human agents looking to participants’ right (0.634 ± 0.016 s) compared to the triangle (p_MLM_< 0.001, |d| = 0.546, BF10 = 1.296e+03). No other comparisons survived Bonferroni correction (p_MLM_> 0.051, p_MultComp_> 0.189, |d| < 0.308, BF10 < 3.112).

#### Interim discussion experiment 4

We observed that trying to infer why an agent is gazing toward an object is faster when people observe humans compared to non-human agents. Participants were faster in detecting humans’ intentions to speak and to grasp compared to the triangle and were faster in detecting humans’ intention to grasp compared to robots. Results were not affected by the participants’ visual familiarity with robots (see Supplementary Information).

The results indicate that anticipating what an agent is going to do may affect how others’ gaze is processed. Indeed, we observed that participants were always faster in detecting human gaze directed toward participants’ right and left compared to the triangle. Note that Experiment 1 showed no differences and Experiment 2 showed a small difference only for the right side of participants (Experiment 2, |d| = 0.447, BF10 = 187.351; Experiment 4, |d| = 0.546, BF10 = 1.296e+03). Moreover, the effect size for the difference between detecting a human compared to a robot’s gaze toward the participant’s left was larger in Experiment 4 than Experiment 1 (Experiment 1, |d| = 0.382, BF10 = 16.533; Experiment 4, |d| = 0.474, BF10 = 1.630e+02).

Interestingly, we observed no difference when the robots were going to speak or were looking toward the participants’ right. Although we did not observe a three-way interaction for RT (Groups by Agent by Gaze), visual inspection of the data (Figure S3) suggests that when the graspable object was located on the right side of the screen (i.e., participants’ right, agents’ left), attributing the intention to speak was faster for humans than robots. On the contrary, RT for the intention to grasp was not influenced by the graspable object location (i.e., RT was faster for humans than other agents). This result may suggest that participants have processed motor and communicative intentions differently depending on the spatial location of the graspable object. That is, the processing time for attributing the intention to speak may have been favored by the proximity of the graspable object to the responding hand. This may suggest a potential pre-activation of the motor system well before the agent appeared and gazed (note that the scenario was displayed for 1100 ms before the agent appeared). However, this potential advantage of the motor system in interpreting a social action was specific for humans and not for non-human agents. Future studies should investigate if motor and social intentions are processed differently.

Notable, we observed that attributing the intentions to grasp and speak took longer for the triangle compared to the simple detection of up|down movements. This further suggests that participants tried to actively reflect upon others mental states rather than using a simple spatial strategy to solve the task.

These results are not in line with what we should have expected if participants were using either an allocentric or an egocentric spatial strategy to solve the task. Thus, the fact that we did not replicate in full any of the findings of experiments 1 and 2 (see also Figure S2 for a graphical visualization of the Experiment 4 data based on where the agent was looking) suggests that participants did not use only a spatial rule to solve the task (e.g., responding uniquely based on where the agent was looking given that right and left gaze movements implied the same intention throughout the whole task). Contrary, findings in Experiment 4 may indicate that after detecting a gaze direction, additional processes responsible for action interpretation and the attribution of motor and communicative intentions to (non-) human agents may have intervened. Nonetheless, it may be possible that participants computed a line of sight to resolve experiments 3 and 4. This explanation may be unlikely because we did not see the response time of Nao to be faster than all other agents (as Nao was close to the gazed objects; Surtees et al., 2013; Michelon and Zacks, 2006; see plots of all the main tasks separated by Agent on the online repository).

Overall, the findings from experiments 3 and 4 suggest that when participants are asked to reflect upon others’ mental content (either what the agent is seeing or is going to do), the interpretation of the observed action is faster for humans compared to non-human agents.

### Experiment 5– Detecting where an agent is looking (vertical axis)

Given the result from previous experiments, we reasoned that if reflecting upon others’ mental content may highlight differences across the agents (while detecting gaze direction change may not), we should achieve similar results in a simpler setup where the only factor that is changed is the axis along which gaze can be directed.

Here, participants were instructed that each agent could look up or down (i.e., we removed the graspable objects and the text bubble), and they were asked to indicate where the agent was looking (up or down; non-mentalistic task demand) using two different orthogonal keys (see STAR Method section). Given the non-mentalistic task we expected no differences across agents and gaze directions. We aimed to collect data from 50 participants and collected 48 individual datasets.

We removed trials with RTs that were deemed too fast or too slow (0.59%). Then, trials with RTs falling above or below 2.5SD of the overall mean within each block of each participant were removed (2.53%). No participants had a performance below 65%. Three participants with a performance (either accuracy or RT) above or below 2.5SD of the overall mean across conditions of the remaining participants were excluded from the final sample. The final sample size for this experiment was n = 45.

#### Main task performance

**Figure 6:**
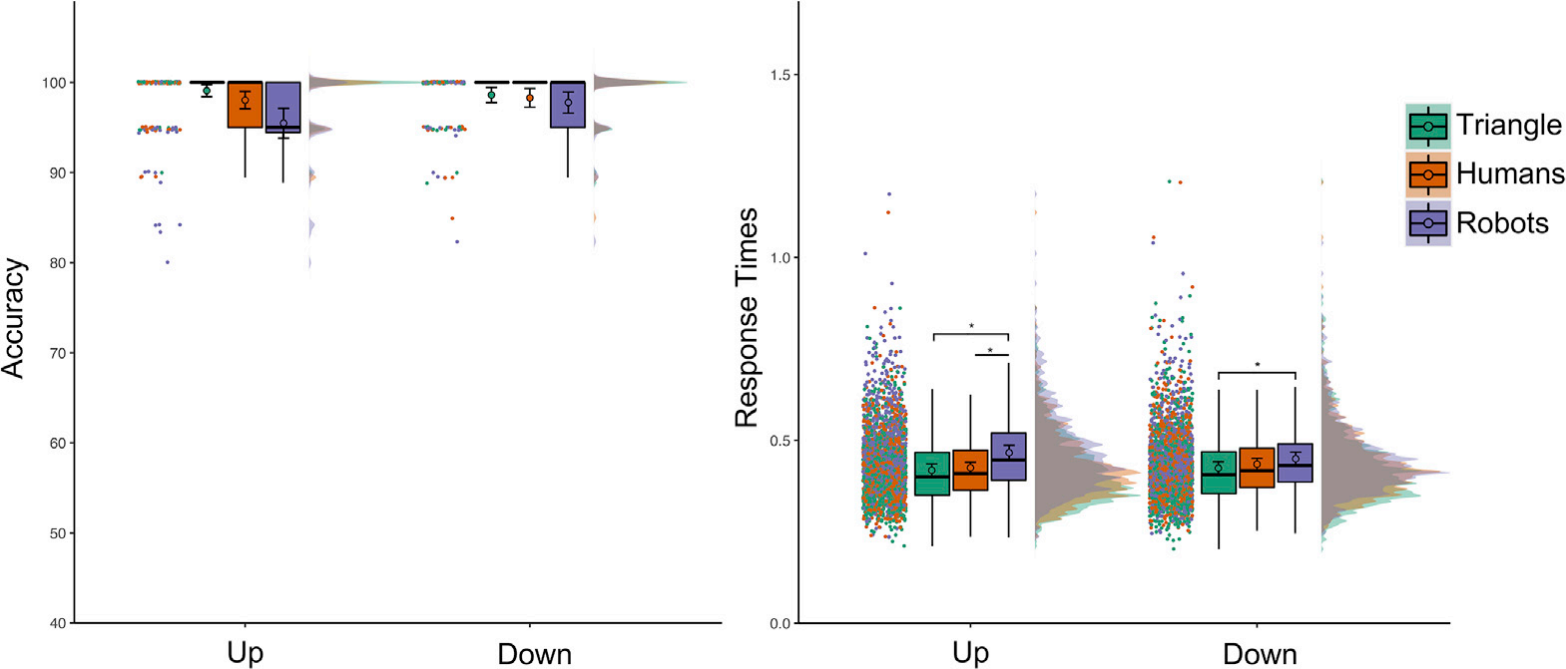
Results of Experiment 5 Accuracy percentage is shown on the left, Response Times expressed in seconds on the right. No interaction between the agents and the observed action was observed for the accuracy measure. For consistency across the figures of all experiments we display accuracy measure for each experimental condition. We invite the reader to refer to the main text for accuracy results. The labels “Up” and “Down” indicate the condition where participants observed the agent looking up and down respectively. See Figure 2 for a detailed explanation of our data visualization approach.

We analyzed performance measures (see Figure 6) with Agent (human, robot, triangle) and Gaze (up, down) as within-subject’s factors.

For accuracy, the effect of Gaze, χ2(2) = 0.457, p_MLM_ = 0.499, and the two-way interaction, χ2(2) = 4.080, p_MLM_ = 0.130, were not significant. We observed a main effect of Agent, χ2(2) = 12.091, p_MLM_ = 0.002, with participants being less accurate in recognizing the gaze of the robots (97.27 ± 0.36%) compared to the triangle (98.58 ± 0.26%; p = 0.002, |d| = 0.400, BF10 = 18.743). Visual inspection of the data suggests that this result was mainly driven by a reduced accuracy in detecting robots looking up compared to the other agents. No other Bonferroni corrected pvalues were lower than 0.05 for both multiple comparisons computed on the estimates of the simplified MLM and multiple comparisons using pairwise t-tests on aggregated data (p_MLM_> 0.117, p_MultComp_> 0.008, |d| < 0.473, BF10 < 12.095).

For RT, we removed incorrect answers (2.10%) from the final dataset. We observed a main effect of Agent, F(2,175.8) = 53.956, p_MLM_< 0.001, ηp2 = 0.385, no effect of Gaze, F(1,44) = 0.006, p_MLM_< 0.939, ηp2 = 0.001, and a significant Agent by Gaze interaction, F(2, 175.9) = 3.938, p_MLM_< 0.001, ηp2 = 0.086. The latter suggested that participants were slower in recognizing the robot looking up (0.466 ± 0.010 s) compared to the triangle (0.418 ± 0.009 s; p_MLM_< 0.001, |d| = 1.239, BF10 = 7.101e+07) and the human (0.425 ± 0.008 s; p_MLM_< 0.001, |d| = 1.156, BF10 = 1.236e+07). The participants were also slower in recognizing the robot looking down (0.450 ± 0.009 s) compared to the triangle (0.424 ± 0.009 s; p_MLM_< 0.001, |d| = 0.646, BF10 = 2.788e+02). No other comparisons survived Bonferroni correction (p_MLM_> 0.055, p_MultComp_> 0.081, |d| < 0.407, BF10 < 4.271).

#### Interim discussion experiment 5

Accuracy and RT did not differ between humans and the triangle. On the contrary, participants made more errors and were slower in detecting the robots looking up compared to the other agents. Results were not affected by the participants’ visual familiarity with robots (see Supplementary Information).

It is possible that while the triangle movements could be solved by simply detecting a change along the vertical axis, participants may have had the impression that agents with eyes (humans and robots) were looking at the microphone placed above their heads. This might have facilitated an automatic perspective-taking of eyed agents (i.e., what is the agent looking at?). Thus, based on experiment 3, participants may have had more difficulty ascribing perceptual content to robots. An alternative explanation is that looking up, which is a typical western-world action people perform when thinking about something (Baron-Cohen et al., 1995; Scherf et al., 2018; McCarthy et al., 2006; Andrist et al., 2014), might have evoked a mentalistic interpretation of human-like agent (not triangles) actions. If this is correct, results would suggest that a mentalistic interpretation of others’ gaze may be less associated with human-like robots. Participants were also slower in detecting robots looking downwards compared to the triangle. This may indicate that the shape of the triangle (pointing downward) was easier to associate with a spatial strategy for recognizing the downward movement. Notably, observing humans and robots did not differ in that condition.

Overall, these results suggest that processing robots’ gaze toward an object required more effort than human gazes and the vertical movements of the triangle. However, despite the non-mentalistic task demand, these results may have implicitly evoked mentalising processes for human-like agents. Hence, in the next and final experiment, we tested whether asking participants to focus on others mental content influences the pattern of results we observed in this experiment.

### Experiment 6– Inferring why an agent is looking at an object (vertical axis)

In this final experiment, participants were instructed that each agent could look down at the table or look up at the microphone to speak. The participants were asked to indicate what the agent was doing (going to speak, looking at the table). If predicting what an agent is going to do is easier for humans, we should expect faster response times for human agents compared to others. We aimed to collect data from 50 participants and collected 47 individual datasets.

We removed trials with too fast or too slow RT (0.89%). Then, trials with RT falling above or below 2.5SD of the overall mean within each block of each participant were removed (2.58%). No participant had a performance below 65%. Finally, two participants had a performance (either accuracy or RT) above or below 2.5SD of the overall mean across conditions of the remaining participants and were excluded (final sample size n = 45).

#### Main task performance

**Figure 7:**
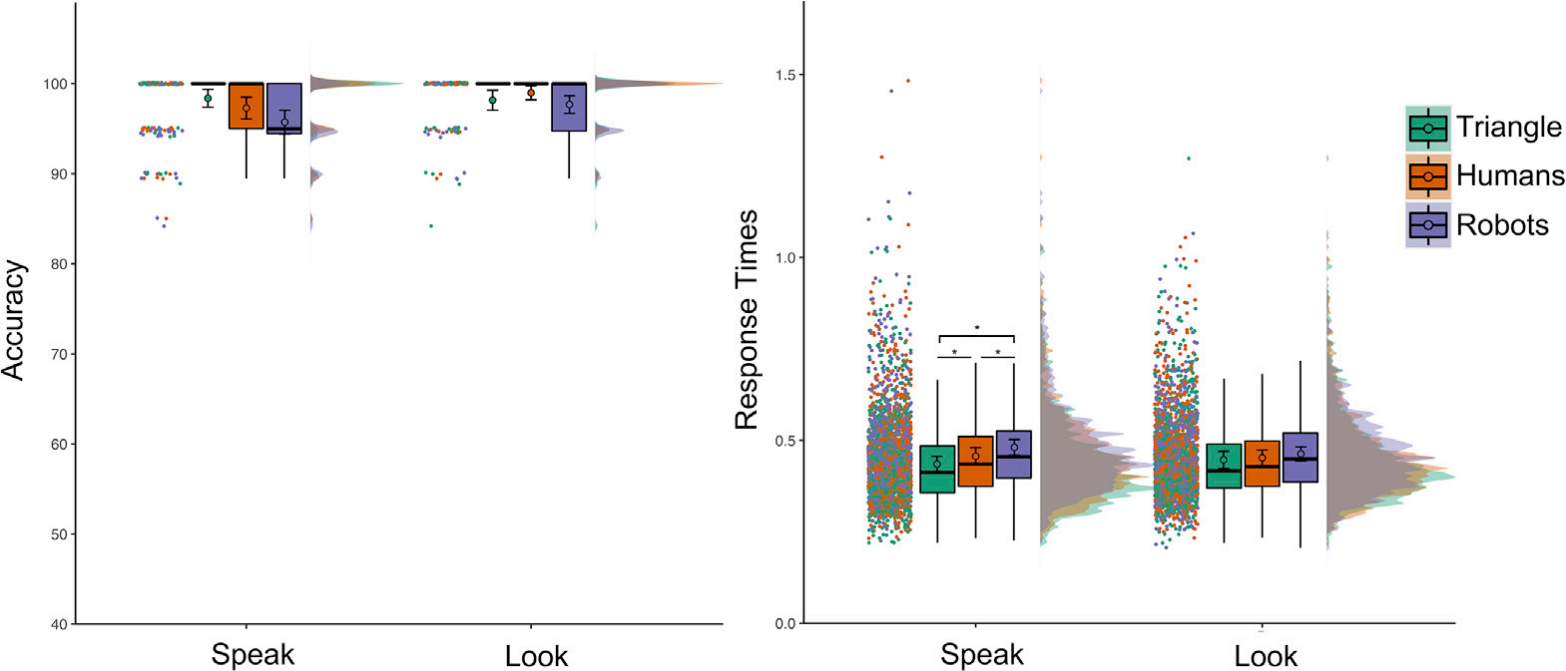
Results of Experiment 6 Accuracy percentage is shown on the left, Response Times expressed in seconds on the right. No interaction between the agents and the observed action was observed for the accuracy measure. For consistency across the figures of all experiments we display accuracy measure for each experimental condition. We invite the reader to refer to the main text for accuracy results. The labels “Speak” and “Table” indicate the condition where participants observed the agent looking up to the microphone to speak and down to the table respectively. See Figure 2 for a detailed explanation of our data visualization approach.

We analyzed accuracy and RT (Figure 7) with Agent (human, robot, triangle) and Intention (speaking, looking at the table) as within-subject’s factors.

For accuracy, we observed a main effect of Agent, χ2(2) = 8.944, p_MLM_ = 0.011, with participants being less accurate in recognizing the gaze of the robots (96.69 ± 0.37%) compared to the triangle (98.26 ± 0.41%; p_MLM_ = 0.041, |d| = 0.416, BF10 = 4.830). Visual inspection of the data suggests that this result was probably driven by a reduced accuracy in detecting robots looking at the microphone. No other Bonferroni corrected pvalues were lower than 0.05 for both multiple comparisons computed on the estimates of the simplified MLM and multiple comparisons using pairwise t-tests on aggregated data (p_MLM_> 0.040, p_MultComp_> 0.056, |d| < 0.364, BF10 < 2.301). We also observed a main effect of Intention (χ2(2) = 4.457, p_MLM_ = 0.027) with participants being more accurate when agents looked at the table (98.27 ± 0.27%) compared to the microphone (97.12 ± 0.38%). The two-way interaction was not significant (χ2(2) = 4213, p_MLM_ = 0.122).

For RT, we removed incorrect answers (2.30%) from the final dataset. We observed a main effect of Agent, F(2,175.8) = 30.694, p_MLM_< 0.001, ηp2 = 0.263, no effect of Intention, F(1,44) = 0.326, p_MLM_ = 0.571, ηp2 = 0.007, and a significant Agent by Intention interaction, F(2, 175.8) = 6.836, p_MLM_ = 0.001, ηp2 = 0.074. The latter suggested that participants were slower in attributing the intention to speak to the robot (i.e., 0.481 ± 0.011 s) compared to the triangle (0.435 ± 0.011 s; p_MLM_< 0.001, |d| = 1.175, BF10 = 1.867e+07) and the human (0.457 ± 0.012 s; p_MLM_< 0.001, |d| = 0.691, BF10 = 6.763e+02). The participants were also slower in attributing the intention to speak to humans compared to the triangle (p_MLM_ = 0.002, |d| = 0.600, BF10 = 1.140e+02). No other Bonferroni corrected pvalues were lower than 0.05 for both multiple comparisons computed on the estimates of the simplified MLM and multiple comparisons using pairwise t-tests on aggregated data (p_MLM_> 0.035, p_MultComp_> 0.171, |d| < 0.363, BF10 < 2.269).

#### Interim discussion experiment 6

The participants made more errors in interpreting robots’ movements compared to the triangle. They were also slower when the robots were looking up to speak compared to humans and the triangle, and increased RTs were observed when attributing humans the intention to speak compared to the triangle. Results were not affected by the participants’ visual familiarity with robots (see Supplementary Information).

We do not think that the observed faster RT for the triangle truly reflects the recruitment of mentalizing processing for non-human-like objects. Rather, the triangle may have been perceived as a simple directional agent rather than an intentional one, and a visual-spatial rather than high-level strategy may have been used to interpret its movements. As such, it may be possible that after observing a spatial change, additional mentalizing processes may have been recruited for interpreting human-like (humans and robots), but not the triangle, movements.

Similarly, the different instructions used in this task (i.e., asking participants to interpret a movement as an action with a purpose) may have led participants to interpret the looking at the table as a non-goal directed action. Hence, no additional processes were required to understand the observed action, which may explain the absence of differences across agents in that condition. Finally, we observed that RT for robots was slower than humans when ascribing the intention to speak. This may further indicate that attributing intentions toward humanoid robots may be more effortful than attributing the same intent to humans.

## Discussion

Understanding others by looking at their gaze is fundamental for human-human and human–robot social interactions. In a series of experiments, participants observed three different agents (humans, human-like robots, and a triangle) gazing and orienting toward different directions and different objects. The participants made explicit judgments about where the agent was looking (experiments 1, 2, and 5), what the agent was looking at (Experiment 3), and why an agent was looking at a specific object (experiments 4 and 6).

The main finding was that interpreting what a human was looking at, or what a human was going to do after gazing, generally required less time compared to other non-human agents. Such an advantage for processing human gaze was clearly observed in tasks requiring participants to represent others’ minds (experiments 3 and 4) rather than tasks focused on detecting a change in others’ gaze (experiments 1-2). This excludes the possibility that people generally perceived robots’ motion and appearance as incongruent (Saygin and Stadler, 2012; Urgen et al., 2018). Moreover, the observed advantage for decoding human perspective and intentions compared to non-human agents cannot be explained merely by spatial accounts, and the mechanisms supporting the ascription of mental content to others may have varied also depending on the observed agent’s visual form. Indeed, processing human gaze was faster than processing robotic gaze (experiments 5-6) but slower than the triangle “gaze” (Experiment 6).

We exclude the interpretation that participants were distracted by the robotic agents because no differences in the control condition were observed in experiments 1—4, and no differences emerged in experiments 5 and 6 between human and robots when they were looking at the table (i.e., not looking at a specific object). Moreover, it is unlikely that the text bubble with mentalistic sentences affected our results (Aliasghari et al., 2021; Mahzoon et al., 2021) because sentences were not linked to a specific agent. Moreover, we exclude that results were affected by the ability to process an object’s location (especially in experiments 4, 5, and 6 where object location did not vary across trials) because we ensured participants had sufficient time to see the scenario (table, graspable objects, text bubble) by presenting it well before the agent appeared.

In the following sections, we discuss how the adopted tasks offer a useful tool to assess people’s ability to represent others’ mental content. We further consider how domain-general cognitive mechanisms cannot explain our data (e.g., participants being generally faster in detecting human gaze compared to other entities). Because we have been able to exclude that participants were simply faster in processing the directional change of the human compared to other agents, our approach also underscores the importance of both control conditions and control agents when assessing mentalizing abilities (Schurz et al., 2015). Finally, we outline the need to go beyond a human-centric approach to study how we perceive and ultimately interact with robots, and move toward an integrated approach that incorporates a fuller representation of the neural and cognitive processes required for supporting successful human-machine interaction (Cross et al., 2016; Henschel et al., 2020; Cross and Ramsey, 2021).

### Does evaluating others gaze provide access to their mental content?

Assessing people’s ability to read others’ minds from gaze observation should rule out low-level explanations (e.g., spatial stimulus-response mapping), and should ensure that an observer is maintaining a distinction between their own and others’ mental states. These two criteria are referred to as the “mentalising” and the “nonmerging” criterion, respectively (Quesque and Rossetti, 2020). We believe that in the current study participants tried to represent others’ mental content. The results showed that human and robot gaze are processed differently when the task required participants to represent an agent’s mental content (experiments 3, 4, and 6) rather than to detect the direction of their gaze (experiments 1, 2 and 5). Moreover, we did not observe any difference between detecting humans and robots gazing toward a spatial location where no object was present (looking up|down in experiments 3 and 4; looking down in experiments 5 and 6). Importantly, to ensure that the detection of a directional change toward a spatial location could not explain our data, we used as a control agent an object with a clear non-biological shape and texture (Schurz et al., 2015), as opposed to an object sharing posture and visual features similar to our human-like robotic agents (Santiesteban et al., 2014). Thus, by using a control condition and a control agent, low-level mechanisms cannot fully explain the results reported in the present study. In this sense, we did not find a general spatial compatibility effect (i.e., faster responses when agents looked to their right, or the correct answer was “right”, as participants responded with the right hand) when participants had to detect where the agent was looking at (experiments 1 and 2). On the contrary, experiments 1 and 2 suggest that the observed actions were more spatially coded for non-human rather than human agents. That is, there may have been a conflict between task- and agent-centred spatial codes and participants’ egocentric frame of reference. For example, when observing the agent looking toward participants’ left in experiment 1 (or participants’ right in experiment2), and the correct answer is “left” while responding with the right hand. Hence, detecting the gaze of a non-human agent (irrespective of its human-like shape) may rely more on visuo-spatial information rather than representing the observed gaze through visual and motor brain areas (e.g., superior temporal sulcus, frontal eye fields; Stephenson et al., 2021). This may explain the observed reduced ability to map the observed action with the correct response for non-human but not human agents in experiments 1 and 2.

It may be argued that a basic associative explanation could explain our data. Indeed, our ability to understand others’ actions can be influenced by our knowledge about an agent (Cross et al., 2016; Bach and Schenke, 2017). For example, learning a person has a preference for kicking a ball will facilitate recognizing that action (but not other actions) whenever the same actor is seen (Schenke et al., 2016). In our study, agents did not have a preferred action, so it is unlikely that any form of implicit learning may explain our results (Heerey and Velani, 2010; Hudson et al., 2012).

Experiment 4 further supports the interpretation that low-level mechanisms and the simple detection of others’ gaze direction are not solely responsible for understanding others’ intentions. Indeed, results did not resemble the findings from the non-mentalistic tasks (experiments 1 and 2) despite the objects’ identity not being relevant to solve the task (i.e., because the objects’ location did not change across trials, the identity of the object, say, located on the participant’s right, was always the same). Similarly, experiments 5 and 6 showed performance differences between humans and robots only when they looked toward an object (i.e., no differences when they looked down). Hence, we suggest that it is plausible that participants solved the tasks by also using high-level social cognition processes.

Nonetheless, it has been suggested that true mentalizing tasks require participants to maintain a clear distinction between self and others’ mental content (Quesque and Rossetti, 2020). This position is strongly based on false belief tasks. Crucially, typical false belief tasks may not reflect our ability to reason upon others mental states but rather the ability to compare different mental representations (Deschrijver and Palmer, 2020). Although the “nonmerging” criterion has been defined as crucial for supporting the claim of mentalizing abilities, we believe this criterion to be more important in text-based and vignette-based tasks where mismatching mental representations between different agents must be detected. Contrary to these text-based setups, online inferences based on observing an agent’s actions may rely more on an automatic non-reflective distinction between self and others. In this sense, we propose that observing others’ gaze and trying to infer their visual perspective or future actions is different from the ability to monitor whether another’s mental state representation is mismatching with one’s own. However, it may still be argued that shared neural mechanisms may make the self/other distinction more difficult when observing others performing an action. It is worth noting that single-cell recording studies showed that not all neurons active during self-generated actions are also active during the observation of actions generated by others (Bonini et al., 2014; Bonini, 2017). Moreover, physiological evidence in humans suggests a potential role of the motor system for an early distinction between self- and other-generated actions (Weiss et al., 2014). Thus, although false belief tasks focus on belief conflict monitoring, we studied the ability to understand others mental content through action observation (i.e., their true belief; Deschrijver and Palmer, 2020).

For all the above reasons, we believe participants did represent the action the observed agent was going to do and that automatic processes at a physiological level may have discriminated the origin of that action (i.e., not self-generated).

### Shifting from human to object social cognition to improve human–robot interaction

It has been proposed that mentalizing abilities can be influenced by the ability to detect others’ gaze direction (Stephenson et al., 2021), and that we may have an advantage in taking the perspective of agents we perceive similar to ourselves (e.g., ingroup vs outgroup; Ye et al., 2021).

The actions participants observed were familiar movements (head and gaze movements for the human and robots), and we reduced the impact of low-level factors (e.g., kinematic differences) by using identical temporal profiles to create agents’ gaze movements. This may have limited the possibility to evoke an ingroup/outgroup categorization of the agent based on human-like or robot-like motor behavior. Importantly, clear differences between human and robotic agents’ performance emerged only when participants were asked to reflect upon their mental states (experiments 3 and 4) compared to non-mentalistic questions (experiments 1-2). This suggests that a solely ingroup-outgroup categorization of the agents cannot fully explain our data. Supporting this, we note that participants did not differ in the control condition where agents moved but did not direct their attention to any object (i.e., up|down condition). Furthermore, as we observed longer RT to encode directional cues toward an object for human and robotic agents compared to the triangle (experiments 5-6), it is unlikely that the saliency and familiarity of face features (both humans and robots had eyes and mouth) can explain our results.

Thus, the fact that performances differed depending on the experimental question (experiments 1, 2, and 5, “Where” taking an egocentric and allocentric perspective for experiments 1 and 2 respectively; Experiment 3, “What”; Experiments 4 and 6, “Why”) and whether the agent looked at an object or not, may suggest that participants represented the agent’s mental content by integrating across multiple sources. A parsimonious interpretation of our data may be that the analysis of both the agent’s visual features (visual bodyform, facial features, and textures) and motor actions, may have differently engaged neural networks attributed to the “social brain” (Henschel et al., 2020; Cross and Ramsey, 2021).

In this respect, gaze and head actions started after the agent was displayed in a static position for 400 ms. This temporal window may have given enough time to process both body and facial features and recruit a wider network of brain areas responsible for integrating motion and semantic information from an observed agent (Quian Quiroga et al., 2008; Harry et al., 2016; Yovel and O’Toole, 2016; Hu et al., 2020). Nonetheless, we showed clear differences between agents only when task instructions required a mentalistic representation of others’ minds. Thus, it is possible that processing and integrating low-level visual features of the observed agent with high-level task demands may have created a mismatch between the qualities expected from human-like bodies (e.g., having a mind, animacy) with their metal-like appearance and slowed the interpretation of robots’ behavior. Supporting this interpretation is the fact that the triangle was generally faster than humans and robots in experiments 5 and 6. This suggests that a visual strategy may have been adopted to detect the triangle’s directional changes in less demanding tasks (compared to experiments 1-4 that required three answers, more attention to process objects’ location, and may have required mental rotations or stimulus-response mapping). Hence, agents with a more human-like appearance may automatically engage mentalizing processes among observers, which are then responsible for matching (or not) the living or non-living nature of the observed agent with typical human qualities (e.g., the capacity to intend and plan). The longer response times for the human-like robots when they gazed toward an object may thus suggest that their visual textures evoking non-living qualities may have slowed the match between the observed action and the underlying intention.

These results hold further relevance for our understanding of how to optimize collaborative physical tasks between humans and robots (e.g., handover tasks) where robots could use gaze cues to communicate the start of a manual reach-to-grasp action. Our findings suggest that when a robot communicates the intention to grasp one object among multiple choices using implicit behavioral cues, trying to infer their intention or to take their perspective may hinder people’s ability to anticipate the robot’s next movement (experiments 3-4). However, results from non-mentalistic tasks suggest that people may understand a robot communicating a spatial location (experiments 1-2), for example, to share the same attentional workspace. This result further corroborates previous studies showing that humans can use spatial information derived from robots’ gaze (Moon et al., 2014).

In summary, our interpretation of the results obtained from six separate experiments is that different visual shapes and textures may evoke specific qualities of the observed agent and consequently recruit a diverse group of neural and cognitive responses within the human social brain networks. Nevertheless, it is essential to note that the recruitment of different brain networks may not indicate a less efficient (in terms of RT and accuracy) processing of the observed stimulus. Instead, they may indicate a distinct qualitative way to understand human and non-human behavior. In this sense, the future of social neuroscience research exploring human–robot interaction, and indeed, the more general concept of human-machine interaction, should aim to extend beyond a human-centric approach to social cognition and explore how an object’s visual appearance interacts with social aspects of perception and interaction.

### Conclusion

We observed that an agent’s visual body-form (human-like vs non-human-like) and visual textures (skin-like versus plastic/metal) may differently affect the processing of high-level social behaviors depending on its human and non-human nature.

Our results cannot be explained by domain-general cognitive mechanisms that simulate the effects of mentalizing in social contexts (Heyes, 2014). Rather, online mindreading (as our tasks required) is likely to rely on the correct integration of the intentional nature of the observed agent (by processing its bodily form and visual textures) with both motor and mentalizing processes during action observation. Moreover, our experimental design and stimuli have introduced important findings and methodological considerations to inform ongoing debates concerning the role of robots’ visual appearance on human collaboration and acceptance (Saygin and Stadler, 2012; Ortenzi et al., 2021; Cross et al., 2016; Mamak, 2021). By using a non-human-like object as control agent and by focusing on gaze behavior, our findings expand existing literature that demonstrated the importance of human-like and robot-like visual textures during the observation and prediction of humanoid robots mechanical manual actions (Saygin et al., 2012; Saygin and Stadler, 2012; Urgen et al., 2018). Further work is now required to examine how implicit signals (e.g., gaze movements) of human-like and non-human-like robots (Micelli et al., 2011; Pan et al., 2018; Sivakumar et al., 2013) facilitate the predictability of artificial agents to positively improve the fluency and subjective experience of human–robot interactions (Ortenzi et al., 2021).

Overall, our findings suggest that ascribing mental content to dynamic displays of agents (what they are looking at, why they are looking to a specific object) differs across human-like and non-human-like artificial agents compared to humans, and may be more difficult for people to achieve when viewing hybrid agents like a human-like robot with machine-like visual features.

### Limitations of the study

Individual perspective-taking abilities are fundamental for social interactions and may have been relevant to solve our tasks. Because of our use of a between-subjects design, we were not able to reliably assess how individual differences in the ability to detect a gaze direction according to an egocentric or allocentric frame of reference may be related to the ability to understand what the agent is doing. One valuable direction for future work will be to try to explore similar questions with within-subjects designs, in order to more convincingly address questions related to individual differences. Moreover, we used only a single self-reported question to account for familiarity with robots. This approach may not have been sensitive enough to highlight differences across participants and does not account for people’s lasting experience in interpreting human faces and bodies. Moreover, we did not assess how participants perceived a mind in our stimuli (Stenzel et al., 2012). Future studies will need to test how individual differences in attributing a mind to a robot may explain our results and how easy is to infer the goals of an agent and which goal the observed action may have evoked (Kupferberg et al., 2018).

Although our conclusion may be limited to screen-based scenarios (Sciutti et al., 2015), results are in line with real-life HRI studies that suggested that human participants may have difficulties in tracking and attributing intentions to robots during action observation (Bisio et al., 2014). However, future studies will need to address how screen-based investigations can be applied in natural settings.

## STAR★Methods

### Key resources table

**Table:** 

REAGENT or RESOURCE	SOURCE	IDENTIFIER
**Deposited Data**		

Dataset and Scripts	This paper	https://osf.io/zq3fg/

**Software and Algorithms**		

R 3.5.1	R-Project	https://www.r-project.org; RRID: SCR_001905
lme4 package (v1.1.27.1)	CRAN	https://cran.r-project.org/web/packages/lme4/index.html; RRID:SCR_015654
effectsize package (v0.4.5)	CRAN	https://cran.r-project.org/web/packages/effectsize/index.html
performance package (v0.7.3)	CRAN	https://cran.r-project.org/web/packages/performance/index.html
MuMIn package (v1.43.17)	CRAN	https://cran.r-project.org/web/packages/MuMIn/index.html
emmeans package (v1.43.17)	CRAN	https://cran.r-project.org/web/packages/emmeans/index.html RRID:SCR_018734
JASP (0.14)	JASP	https://jasp-stats.org; RRID:SCR_015823
Psychopy3	Psychopy	https://www.psychopy.org; RRID:SCR_006571
G∗Power	Heinrich Heine Universität Düsseldorf	http://www.gpower.hhu.de/; RRID:SCR_013726

### Resource availability

#### Lead contact

Further information and requests for resources should be directed to and will be fulfilled by the Lead Contact, Emmanuele Tidoni (e.tidoni@hull.ac.uk).

#### Materials availability

This study did not generate new unique reagents.

### Experimental model and subject details

#### Participants

We recruited adult English-speaking participants via the online research participation platform Prolific Academic (Palan and Schitter, 2018). The task, procedure, and methodology were reviewed and approved by the institutional review boards of the University of Hull (protocol number: FHS150) and carried out in accordance with the standards set by the Declaration of Helsinki. All participants were naïve to the task and purpose of the experiment. Each participant completed a single experiment (we used the ‘excluded participants from previous studies’ screener available in the participation platform) in exchange for monetary compensation and informed consent was obtained before starting the task.

A total of 455 participants completed different online experiments (Experiment 1, number of participants n = 81 [female = 36, male = 45, prefer not to say = 0]; mean age ±s.e.m. 25.93 ± 0.78, range [18-50]; Experiment 2, n = 96 [female = 42, male = 54, prefer not to say = 0]; 27.59 ± 1.19 [18-72]; Experiment 3, n = 101 [female = 40, male = 60, prefer not to say = 1], 25.81 ± 0.87 [18-60]; Experiment 4, n = 82 [female = 29, male = 52, prefer not to say = 1], 26.16 ± 1.08 [18-66]; Experiment 5, n = 48 [female = 17, male = 30, prefer not to say = 1], 24.55 ± 1.16 [18-53]; Experiment 6, n = 47 [female = 17, male = 30, prefer not to say = 0], 24.04 ± 0.75 [18-41]). A sample size calculation (G∗Power; Faul et al., 2007) was performed to have sufficient power to detect small effect sizes for Experiments 2 and 3 (dz = 0.30, Alpha = 0.05, Beta = 0.80, minimum sample size of 90), and Experiments 1 and 4 (dz = 0.35, Alpha = 0.05, Beta = 0.80, minimum sample size of 67). As we observed medium to large effects in Experiments 2, 3, and 4, sample size for Experiments 5 and 6 was computed and updated accordingly (dz = 0.45, Alpha = 0.05, Beta = 0.80, minimum sample size of 41). Anticipating that some participants might be removed (e.g., outliers), we slightly increased the target sample size in all studies to avoid reduced statistical power.

### Method details

#### Procedure

All experiments were performed online. Participants were invited to read the information sheet and communicate any questions to the experimenter if needed. After providing informed consent, participants read the experimental instructions. Three agents were presented and described as humans, robots, and a triangle capable of performing three actions, and the trial timeline was explained (see Video S1 for instructions and trial timeline). After that, participants performed a quick online practice session of 18 trials and received accuracy feedback after their answers for the first 12 practice trials (“CORRECT!” for correct answers; “WRONG! The correct key was [*key label*]! The agent is [*text message that explained what the agent was doing based on experiment instructions*]”, for example “looking to your right” in Experiment 1). After the online practice session, participants started four experimental blocks. Each block comprised 45 trials for Experiments 1-4 (total of 180 trials) and 30 trials for Experiments 5-6 (total of 120 trials). After the main task, participants rated their exposure to media robotic content (“How often do you watch movies, TV series, or play videogames where robots are involved?”) using a nominal scale (1 = Never, 2 = Once every Year, 3 = Once every 6 months, 4 = Once every 3 months, 5 = Once every month, 6 = More than once every month). After the experiment, participants were debriefed as to the purpose of the experiment.

#### Apparatus and task

In Experiments 1-4, each agent could gaze towards a graspable object, a text bubble, or up and down for 20 trials (a total of 60 trials per agent; 180 trials for the whole experiment). We displayed 3D printed geometrical shapes as graspable object (i.e., cube, cylinder, sphere, and a rectangle) and the text bubble could contain one of ten short self-descriptive sentences (i.e., I think, I plan, I desire, I judge, I worry, I believe, I imagine, I relax, I feel, I like). These short sentences were selected to facilitate both physical and mental states attribution to the observed agent (Tamir et al., 2016). Sentences were not associated to any agent and were randomly presented each trial. Moreover, the location of the graspable object and text bubble was randomly generated each trial (with exception of Experiment 4). Thus, while an agent gazed towards the graspable object 20 times, the graspable object was located to the agent’s right or to the agent’s left randomly (e.g., an agent could gaze at the graspable object located on their left 12 times, and 8 times at the graspable object located on their right). This randomisation was preferred over counterbalancing as we were interested in testing differences across agents when they were looking towards objects (Experiment 3 was completed first). We decided to keep the same randomisation procedure for Experiments 1 and 2 to make comparable analyses across Experiments 1, 2, and 3 (the main analyses of Experiments 1-2 and the “role of Gaze” analyses of Experiment 3). Participants were asked to place their right index, middle, and ring fingers over three keys (‘n’, ‘j’, ‘i’) and to indicate as fast and as accurate as possible where the agent was from an egocentric perspective (e.g., the agent is looking to his right; Experiment 1), from an allocentric perspective (e.g., the agent is looking to your right; Experiment 2), to indicate what the agent was looking at (e.g., the agent is looking at the object; Experiment 3), or what the agent was going to do (e.g., the agent is looking at the object to grasp; Experiment 4).

In Experiments 5-6, each agent could direct their gaze up towards a microphone placed over their heads (20 trials) or down (20 trials) for a total 120 trials. Participants were asked to place their right index, and middle fingers over two keys (‘l’, ‘k’) and to indicate as fast and as accurate as possible where the agent was looking (e.g., up or down; Experiment 5), or what was going to do (e.g., the agent is going to speak or is looking at the table; Experiment 6).

Keys were randomly assigned to one gaze direction (Experiments 1, 2, and 5) or action (Experiments 3, 4, and 6) across participants. In Experiments 1-4, the up and down gaze movements were associated to the same (randomised across participants) key.

Trial timeline was identical in all experiments. Participants observed for 1100ms a picture of the table with either a graspable object on one side and a text bubble on the other side in Experiments 1-4, or a microphone at the top in Experiments 5-6. Then, one of the three agents could appear, and 400ms later they turned their head and gaze towards one of the presented objects (graspable objects and text bubble for Experiments1-4; a microphone for Experiments 5-6) or not (either up or down for Experiments 1-4; down for Experiments 5-6). The agent and the environment remained on screen until keypress. In all experiments the intertrial interval randomly ranged between 400ms and 600ms. The tasks were developed using Psychopy3 (Peirce et al., 2019) and were hosted on Pavlovia (Pavlovia.org).

### Quantification and statistical analysis

We collected task Accuracy and Response Time (expressed in seconds) as performance measures, and we specified how data would be processed in the online pre-registration file for Experiment 3, the first experiment we conducted (AsPredicted; 54499: https://aspredicted.org/nq822.pdf). Specifically, for all experiments we excluded trials <0.150 s and >1.500 s.. Although, we pre-registered to exclude trials <0.150 sec and >3.000 sec, we preferred to adopt a more stringent criteria in the paper to avoid data to be driven by very long RT by excluding trials >1.500 sec (Bayliss and Tipper, 2005; Wykowska et al., 2014; Kompatsiari et al., 2018). This allowed us to remove motor responses anticipating gaze onset or influenced by non-controllable factors (e.g., participants getting distracted by surrounding noise). Then, for each participant, we excluded trials whose RTs fell above or below 2.5 SDs of the overall mean within each block. At this stage of data processing, we excluded participants whose overall accuracy was below 65%. Although this value was arbitrary, it is well above chance level for Experiments 1, 2, 3 and 4 (33%) and Experiment 5-6 (50%). Hence, participants randomly responding should have been excluded from the final sample. Finally, we excluded participants considered outliers when their performance (in RTs or Accuracy) fell above or below 2.5 SDs of the overall mean across conditions of the remaining participants.

On the final dataset, statistics were performed using R 3.5.1 (R Core Team, 2018) run on the University of Hull High-Performance facility VIPER (http://hpc.wordpress.hull.ac.uk/home/). We used the lme4 package (v1.1.27.1; Bates et al., 2015) to perform MLM with fixed effects and complex random intercepts (CRIs) as scalar random effects (Scandola and Tidoni, 2021). Scalar random effects can represent the complexity of categorical factors (e.g., in lme4 syntax 1 | Participants:Factor; Factor; Bates et al., 2015). Model reduction started from the full-CRIs MLM (Scandola and Tidoni, 2021) with all main effects and interaction of interests. If the model overfitted, the CRI with the lowest variance was removed until a convergent non-singular model was found. For MLMs on RT of correct answers we also report the partial eta-squared as a measure of effect size (effectsize v0.4.5; Ben-Shachar et al., 2020). For all MLM we computed the conditional R2 (for lme4::lmer performance v0.7.3, Lüdecke et al., 2020; for lme4::glmer MuMIn v 1.43.17, Kamil, 2016). Throughout the paper we report the p-values computed on the estimates of the simplified MLM, and for each multiple comparison we report the individual Bonferroni corrected p-values computed from the final MLM using emmeans (Lenth et al., 2020). Furthermore, we performed confirmatory ANOVAs on mean-aggregated data to support the main analyses. For each confirmatory analyses we ran multiple comparisons on the mean-aggregated data (Scandola and Tidoni, 2021) and report the absolute value of the Cohen’s d (|d|) and the Bayes Factor (BF10; default Cauchy prior of 0.707; JASP Team, 2021, Version 0.14) to further facilitate the reader in assessing the strength of the evidence. Classically, BF10 is interpreted as showing very strong evidence towards the alternative hypothesis when greater than 150, strong evidence when equal or greater than 20, positive evidence when equal or greater than 3, and with weak or negligible evidence when between 1 and 3 (Raftery, 1995). The inverse of these values (1/150, 1/20, 1/3) can be interpreted as BF10 showing very strong, strong, or positive evidence towards the null hypothesis.

We considered as non-conclusive results discordant findings obtained from the MLM and from the analyses on mean-aggregated data. If not stated otherwise, the ANOVAs and multiple comparisons performed on mean-aggregated data confirmed the results obtained from the MLM model.

A multinomial test assessed that the answers to the robotic content exposure question were equally distributed across the 6 options (see Supplementary Information).

### Additional resources

Pre-registration file for Experiment3: https://aspredicted.org/nq822.pdf.

## Data Availability

The datasets generated during this study are available at the Open Science Framework Repository: https://osf.io/zq3fg/. All original code are available at the Open Science Framework Repository: https://osf.io/zq3fg/. Any additional information required to reanalyze the data reported in this paper is available from the lead contact upon request.
