# Supplementary material for: Human but not robotic gaze facilitates action prediction
Source: iScience. 2022 May 25;25(6):104462. doi: 10.1016/j.isci.2022.104462 (PMC9189121; doi:10.1016/j.isci.2022.104462)
Supplement: Document S1.Figures S1–S3, Table S1 and Supplementary Information [file mmc1.pdf]

iScience, Volume *25*

Supplemental information

Human but not robotic gaze

facilitates action prediction

Emmanuele Tidoni, Henning Holle, Michele Scandola, Igor Schindler, Loron Hill, and Emily S. Cross

Supplementary Information

The role of Visual Familiarity

Participants rated their exposure to media robotic content (“How often do you watch movies, TV series, or play videogames where robots are involved?”) using a nominal scale (1= Never, 2= Once every Year, 3= Once every 6 months, 4= Once every 3 months, 5= Once every month, 6= More than once every month). For all experiments we divided the final sample in two groups based on the reported visual familiarity. In particular, we categorised answers higher than 4 as high exposure, and answers equal to or lower than 4 as low exposure. We thought that being exposed 12 times or more per year was an adequate cut-off to separate high and low exposure. This approach also revealed to be adequate to have two groups of similar size.

Experiment 1

There were differences in robotic media exposure (assuming equal proportions of 12; $\chi^2(5) = 12.757$, $p = 0.026$). To further control any role of visual familiarity (VM) we analysed performance measures with participants who reported high and low levels of robotic media content exposure as between-subjects’ factors (Low-Media = 39; High-Media = 35).

For accuracy, we observed no main effect of VM, and no interaction of VM with the two main effects, $\chi^2 < 5.116$, $p_{MLM} > 0.077$.

For RT, we observed a VM by Gaze interaction, $F(2, 144.1) = 3.975$, $p_{MLM} = 0.021$, $\eta p2 = 0.052$. However, multiple comparisons did not reveal any difference ($p_{MLM} > 0.059$, $p_{MultComp} > 0.107$, $|d| < 0.421$, $BF_{10} < 2.630$). We observed a triple interaction in the MLM model, $F(4, 286.2) = 2.569$, $p_{MLM} = 0.038$, $\eta p2 = 0.037$, not confirmed on aggregated data analysis, $F(4, 288) = 2.158$, $p_{Anova} = 0.074$, $\eta p2 = 0.029$.

Experiment 2

There were no differences in robotic media exposure (assuming equal proportions of 14; $\chi^2(5) =$

8.857, $p = 0.087$). To further control any role of VM, we performed an ANOVA on performance measures with participants who reported high and low levels of robotic media content exposure as between-subjects' factors (Low-Media = 45; High-Media = 38).

For accuracy, we observed no main effects of VM, no interaction with the two main effects, $\chi^2 < 1.335$, $p_{MLM} > 0.512$.

For RT, we observed no main effect of VM, no interaction with the two main effects, $F < 1.111$, $p_{MLM} > 0.345$, ηp2 < 0.010.

Experiment 3

There were differences in robotic media exposure (assuming equal proportions of 16; $\chi^2(5) = 47.168$, $p < 0.001$). To further control any role of VM, we performed a control ANOVA on performance measures with participants who reported high and low levels of media content exposure as a between-subjects factor (Low-Media = 39; High-Media = 56).

For accuracy, we observed a main effect of VM, $\chi^2(1) = 3.887$, $p_{MLM} = 0.049$, with participants being more accurate in the low- (97.73 ± 0.23%) compared to the high-media exposure (96.39 ± 0.30%) group. No interactions with the two main effects, $\chi^2 < 4.057$, $p_{MLM} > 0.399$, were observed.

For RT, we observed no main effects of VM, and no interactions with the two main effects, $F < 1.827$, $p_{MLM} > 0.164$, ηp2 < 0.020.

Experiment 4

There were no differences in robotic media exposure (assuming equal proportions of 12; $\chi^2(5) = 10.096$, $p = 0.073$). To further control any role of VM, we performed a control ANOVA on performance measures with participants who reported high and low levels of media content exposure as a between-subjects factor (Low-Media = 44; High-Media = 29).

For accuracy, we observed no main effects of VM, no interaction with the two main effects,

$\chi^2 < 2.720$, $p_{MLM} > 0.099$.

For RT, we observed no main effects of VM, no interaction with the other three main effects, $F < 1.746$, $p_{MLM} > 0.140$, $\eta p2 < 0.025$.

Experiment 5

There were no differences in robotic media exposure (assuming equal proportions of 8; $\chi2(5) = 6.066$, $p = 0.299$). To further control any role of VM, we performed a control ANOVA on performance measures with participants who reported high and low levels of media content exposure as a between-subjects factor (Low-Media = 23; High-Media = 22).

For accuracy, we observed no main effects of VM, and no interaction with the two main effects, $\chi2 < 2.166$, $p_{MLM} > 0.141$.

For RT, we observed no main effect of VM, no interaction with the two main effects, $F < 1.497$, $p_{MLM} > 0.227$, $\eta p2 < 0.017$.

Experiment 6

There was a difference in robotic media exposure (assuming equal proportions of 8; $\chi2(5) = 14.333$, $p_{MLM} = 0.014$. To further control any role of VM, we performed an ANOVA with participants who reported high and low levels of media content exposure as a between-subjects factor (Low-Media = 21; High-Media = 24).

For accuracy, we observed no main effects of VM, no interaction with the two main effects, $\chi2 < 0.526$, $p_{MLM} > 0.468$.

For RT, we observed no main effect of VM, no interaction with the two main effects, $F < 2.349$, $p_{MLM} > 0.098$, $\eta p2 < 0.027$.

Figure S1. Stimuli Creation, Related to Figure 1.

On the left we present the picture taken on Baxter. On the right, we show the edited image with transparent background.

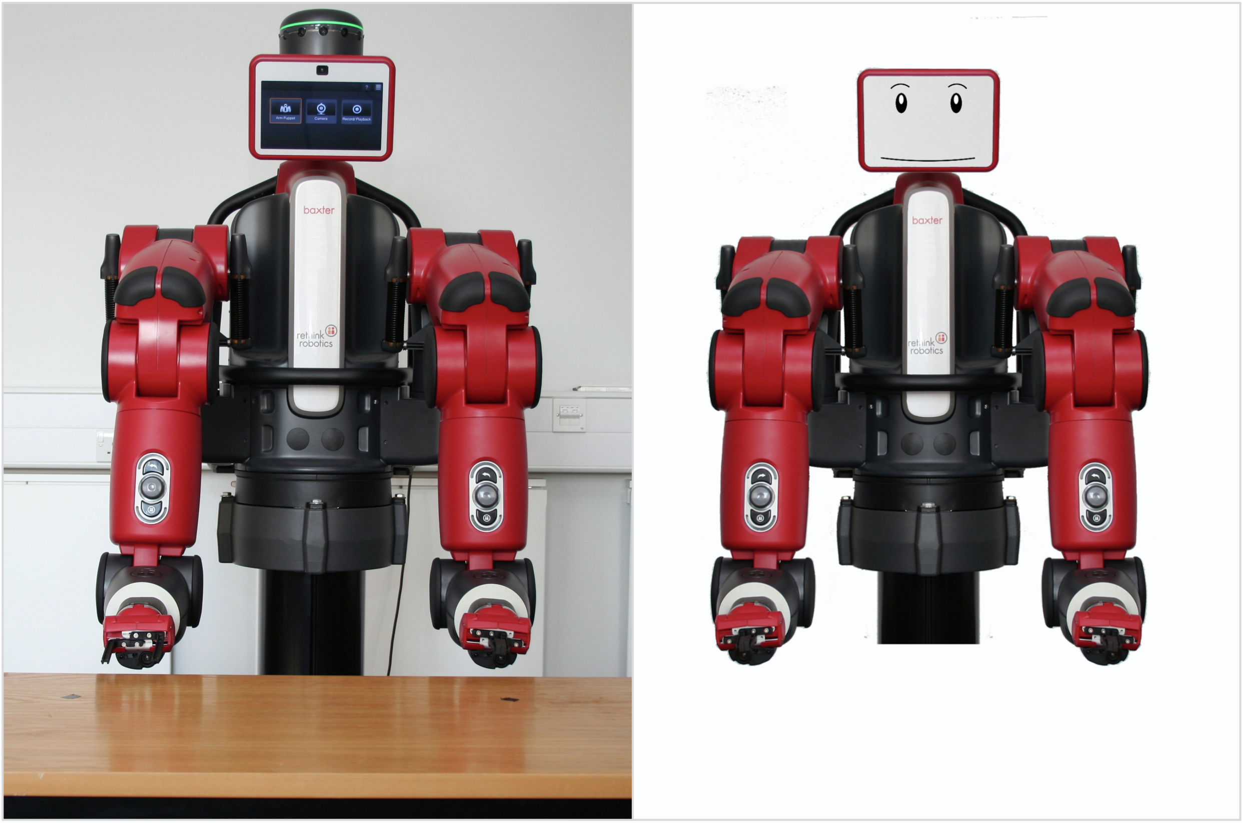

Figure S2. Experiment 4 Results (by Gaze direction), Related to Figure 5.

Graphical representation of Experiment 4 performance based on where the agent was looking at (participant's left, right, or up|down). The reader is invited to refer to Figure 2 in the main text for a detailed explanation of our data visualisation approach.

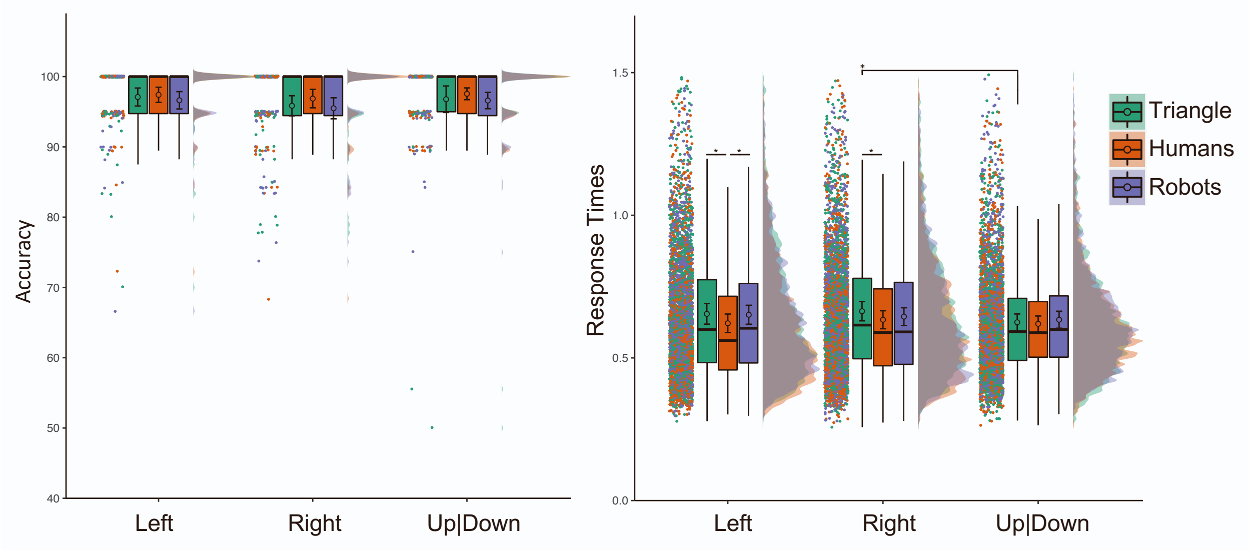

Figure S3. Graphical representation of Experiment 4 by Object location, Related to Figure 5.

Attributing the intention to grasp was not affected by objects location. Contrary, the absence of a difference in attributing a communicative intention to humans and robots (i.e., the average between the intention to speak when the graspable object was located on the participant's right and on the participant's left) may have been driven mainly by the absence of difference when the text bubble was located on the participant's right (i.e., the graspable object was located on the participant's left). In other words, participants took the same amount of time to attribute the intention to speak to humans and robots when the observed agents looked towards the participant's right and the graspable object was far from their responding right hand. The reader is invited to refer to Figure 2 in the main text for a detailed explanation of our data visualisation approach.

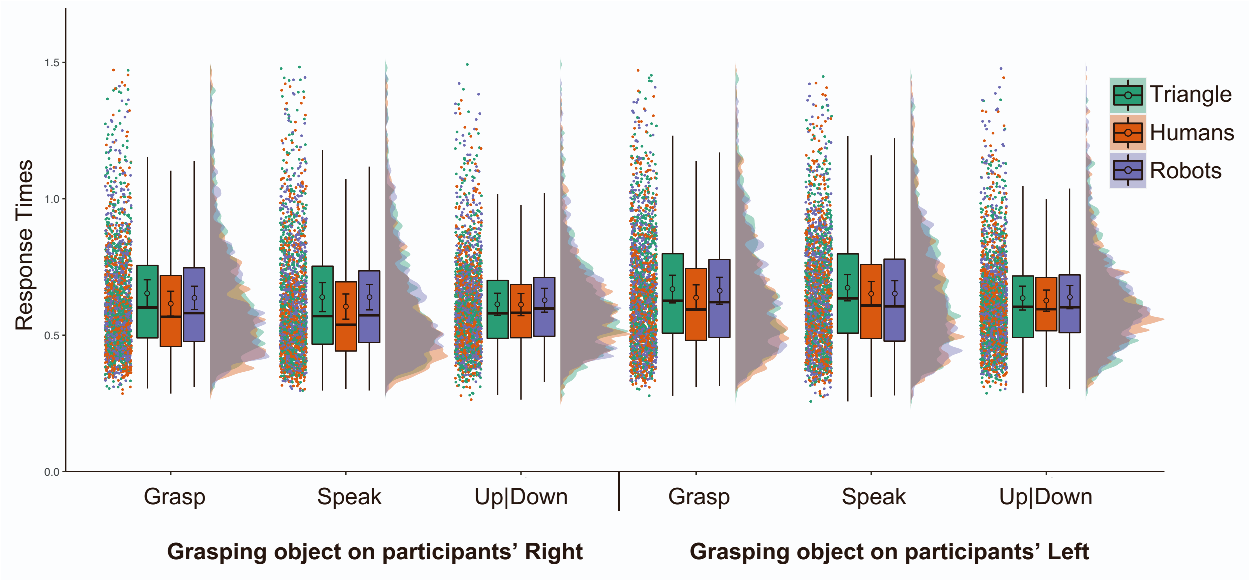

Table S1. Mixed Models for Accuracy and RT, related to QUANTIFICATION AND STATISTICAL ANALYSIS.

The fixed and random effects structure of all analyses for each experiment. Scaled predictors were: the age of participants (Age), the visual familiarity with robots (Fam), the trial number corresponding to each data entry for each participant (nTrial). Categorical predictors were: the location of the objects (Scene; two levels: if the graspable object/text bubble was on the right/left or left/right of the screen), the categorisation of the participant's visual familiarity with robots (FamCat; two levels: low or high), the agents observed by participants (Agent; three levels: Humans, Robots, Triangle), the direction of the agent's gaze (Att; three levels: towards its left, right, up or down), the object the agent looked at (Int; three levels: towards the graspable object, the text bubble, or up|down). For each model we indicate if an optimiser was necessary to achieve a convergent and non-singular model. Finally, we also indicate the Conditional R2, ranging from 0 to 1, to describe the proportion of variance explained by both the fixed and random factors.

ACCURACY

Analyses – Experiment		Fixed Effects	Random Effects	Optimizer	Conditional R2
Main Task	1	Age+Fam+Agent*Att	(1|Subj)+(1|Subj:Att)+(1|Subj:Agent:Att)	✓	0.398
	2	Age+Fam+Agent*Att	(1|Subj)+(1|Subj:Att)+(1|Subj:Agent:Att)	✓	0.487
	3	Age+Fam+Agent*Int	(1|Subj)+(1|Subj:Agent)+(1|Subj:Int)+(1|Subj:Agent:Int)	✓	0.386
	4	Age+Fam+Scene*Agent*Int	(1|Subj)+(1|Subj:Int)+(1|Subj:Agent:Int)	✓	0.409
	5	Age+Fam+Agent*Att	(1|Subj)+(1|Subj:Agent)+(1|Subj:Agent:Att)	✓	0.264
	6	Age+Fam+Agent*Int	(1|Subj:Agent)+(1|Subj:Int)+(1|Subj:Agent:Int)	✓	0.208

Visual Familiarity	1	Age+FamCat*Agent*Att	(1|Subj)+(1|Subj:Att)+(1|Subj:Agent:Att)	✓	0.402
	2	Age+FamCat*Agent*Att	(1|Subj)+(1|Subj:Att)+(1|Subj:Agent:Att)	✓	0.487
	3	Age+FamCat*Agent*Int	(1|Subj)+(1|Subj:Agent)+(1|Subj:Int)+(1|Subj:Agent:Int)	✓	0.384
	4	Age+Scene+FamCat*Agent*Int	(1|Subj)+(1|Subj:Int)+(1|Subj:Agent:Int)	✓	0.409
	5	Age+FamCat*Agent*Att	(1|Subj)+(1|Subj:Agent)+(1|Subj:Agent:Att)	✓	0.254
	6	Age+FamCat*Agent*Int	(1|Subj)+(1|Subj:Agent)+(1|Subj:Int)+(1|Subj:Agent:Int)	✓	0.211
Looked-At Object	1	Age+Fam+Agent*Int	(1|Subj)+(1|Subj:Int)+(1|Subj:Agent:Int)	✓	0.367
	2	Age+Fam+Agent*Int	(1|Subj)+(1|Subj:Int)+(1|Subj:Agent)	✓	0.448
Attention	3	Age+Fam+Agent*Att	(1|Subj)+(1|Subj:Agent)+(1|Subj:Att)+(1|Subj:Agent:Att)	✓	0.372
	4	Age+Fam+Scene*Agent*Att	(1|Subj)+(1|Subj:Att)+(1|Subj:Agent:Att)	✓	0.409

RESPONSE TIMES

Analyses – Experiment		Fixed Effects	Random Effects	Optimizer	Conditional R2
Main task	1	nTrial+Age+Fam+Scene+Agent*Att	(1|Subj)+(1|Subj:Agent)+(1|Subj:Att)+(1|Subj:Agent:Att)		0.396
	2	nTrial+Age+Fam+Scene+Agent*Att	(1|Subj)+(1|Subj:Att)+(1|Subj:Agent:Att)		0.425
	3	nTrial+Age+Fam+Scene+Agent*Int	(1|Subj)+(1|Subj:Agent)+(1|Subj:Int)+(1|Subj:Agent:Int)	✓	0.351
	4	nTrial+Age+Fam+Scene*Agent*Int	(1|Subj)+(1|Subj:Agent)+(1|Subj:Int)+(1|Subj:Agent:Int)		0.437
	5	nTrial+Age+Fam+Agent*Att	(1|Subj)+(1|Subj:Att)+(1|Subj:Agent:Att)		0.342
	6	nTrial+Age+Fam+Agent*Int	(1|Subj)+(1|Subj:Int)+(1|Subj:Agent:Int)		0.356
	1	nTrial+Age+Scene+FamCat*Agent*Att	(1|Subj)+(1|Subj:Agent)+(1|Subj:Att)+(1|Subj:Agent:Att)	✓	0.397

	2	nTrial+Age+Scene+FamCat*Agent*Att	(1|Subj)+(1|Subj:Att)+(1|Subj:Agent:Att)		0.425
	3	nTrial+Age+Scene+FamCat*Agent*Int	(1|Subj)+(1|Subj:Agent)+(1|Subj:Int)+(1|Subj:Agent:Int)		0.351
Visual Familiarity	4	nTrial+Age+Scene+FamCat*Agent*Int	(1|Subj)+(1|Subj:Agent)+(1|Subj:Int)+(1|Subj:Agent:Int)		0.437
	5	nTrial+Age+FamCat*Agent*Att	(1|Subj)+(1|Subj:Att)+(1|Subj:Agent:Att)		0.343
	6	nTrial+Age+FamCat*Agent*Int	(1|Subj)+(1|Subj:Int)+(1|Subj:Agent:Int)	✓	0.357
Looked-At Object	1	nTrial+Age+Fam+Scene+Agent*Int	(1|Subj)+(1|Subj:Agent)+(1|Subj:Int)+(1|Subj:Agent:Int)		0.383
	2	nTrial+Age+Fam+Scene+Agent*Int	(1|Subj)+(1|Subj:Int)+(1|Subj:Agent:Int)		0.412
Attention	3	nTrial+Age+Fam+Scene+Agent*Att	(1|Subj)+(1|Subj:Agent)+(1|Subj:Att)+(1|Subj:Agent:Att)		0.339
	4	nTrial+Age+Fam+Scene*Agent*Att	(1|Subj)+(1|Subj:Agent)+(1|Subj:Att)+(1|Subj:Agent:Att)		0.437
